# Rbm20 antisense oligonucleotides alleviate diastolic dysfunction in a mouse model of cardiometabolic heart failure (HFpEF)

**DOI:** 10.1093/cvr/cvaf171

**Published:** 2025-10-17

**Authors:** Mei Methawasin, Stefan Meinke, Michael H Radke, Gerrie P Farman, Zaynab Hourani, John E Smith, Wei Guo, Henk Granzier, Michael Gotthardt

**Affiliations:** Cellular and Molecular Medicine and Sarver Molecular Cardiovascular Research Program, University of Arizona, 1656 E Mabel ST, MRB 340, Tucson, AZ 85724, USA; Medical Pharmacology and Physiology, University of Missouri, MA415 Medical Science Building, Columbia, MO 65212, USA; Translational Cardiology and Functional Genomics, Max Delbrück Center for Molecular Medicine in the Helmholtz Association, Robert Rössle Str. 10, Berlin 13125, Germany; German Center for Cardiovascular Research (DZHK), Partner Site Berlin, Berlin 10785, Germany; Translational Cardiology and Functional Genomics, Max Delbrück Center for Molecular Medicine in the Helmholtz Association, Robert Rössle Str. 10, Berlin 13125, Germany; German Center for Cardiovascular Research (DZHK), Partner Site Berlin, Berlin 10785, Germany; Cellular and Molecular Medicine and Sarver Molecular Cardiovascular Research Program, University of Arizona, 1656 E Mabel ST, MRB 340, Tucson, AZ 85724, USA; Cellular and Molecular Medicine and Sarver Molecular Cardiovascular Research Program, University of Arizona, 1656 E Mabel ST, MRB 340, Tucson, AZ 85724, USA; Cellular and Molecular Medicine and Sarver Molecular Cardiovascular Research Program, University of Arizona, 1656 E Mabel ST, MRB 340, Tucson, AZ 85724, USA; Department of Animal and Dairy Sciences, University of Wisconsin-Madison, Madison, WI 53706, USA; Cardiovascular Research Center, University of Wisconsin-Madison, Madison, WI 53706, USA; Cellular and Molecular Medicine and Sarver Molecular Cardiovascular Research Program, University of Arizona, 1656 E Mabel ST, MRB 340, Tucson, AZ 85724, USA; Translational Cardiology and Functional Genomics, Max Delbrück Center for Molecular Medicine in the Helmholtz Association, Robert Rössle Str. 10, Berlin 13125, Germany; German Center for Cardiovascular Research (DZHK), Partner Site Berlin, Berlin 10785, Germany; Department of Cardiology, Charité Universitätsmedizin Berlin, Berlin 10115, Germany

**Keywords:** HFpEF, RBM20, Titin, ASO, Therapy

## Abstract

**Aims:**

Heart failure with preserved ejection fraction (HFpEF) is prevalent, deadly, and difficult to treat. Risk factors such as obesity and hypertension contribute to cardiac inflammation, metabolic defects, and pathological remodelling that impair ventricular filling in diastole. Titin based stiffness is a main determinant of diastolic function and can be adjusted by the splicing regulator *RNA binding motif protein 20* (RBM20). Inhibition of RBM20 using antisense oligonucleotides (ASOs) induces expression of compliant titin isoforms, which reduce stiffness. However, dose finding and documenting utility in primarily cardiometabolic disease remains challenging.

**Methods and results:**

Here, we optimized RBM20-ASO dosing in a HFpEF mouse model that closely mimics human disease, characterized by metabolic syndrome and comorbidities, but without primary defects in titin or RBM20. Partial inhibition of RBM20 (∼50%) selectively increased compliant titin isoforms, improving diastolic function while preserving systolic performance. This intervention reduced left ventricular stiffness, enhanced relaxation, and mitigated cardiac hypertrophy, despite ongoing systemic comorbidities.

**Conclusion:**

Our findings demonstrate that targeting titin stiffness with Rbm20-ASOs can serve as an alternative or adjunctive therapeutic strategy for HFpEF to restore cardiac function and prevent further organ damage. The approach may offer benefits even in the presence of phenotypic heterogeneity and unresolved systemic comorbidities.

## Graphical Abstract

**Graphical Abstract:**
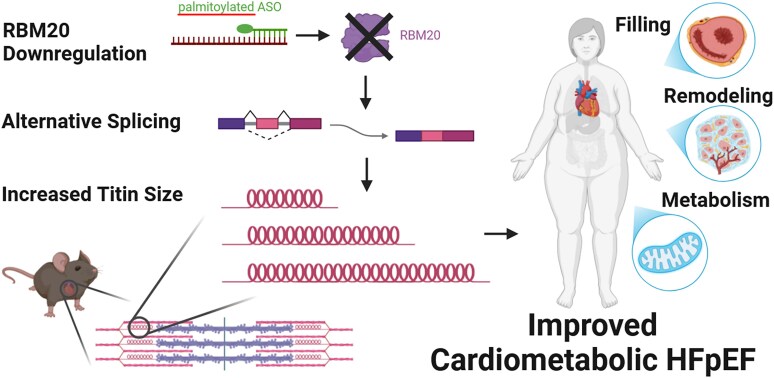

**Time of primary review: 50 days**

## 1. Introduction

Beyond the heart, HFpEF involves complex interactions between various organs and systems, including the lungs, kidneys, skeletal muscle, and vasculature.^1–4^ Systemic inflammation plays a central role, often driven by comorbidities such as obesity, diabetes, and hypertension, which contribute to endothelial dysfunction, impaired nitric oxide signalling, and increased oxidative stress.^5,6^ These factors collectively lead to myocardial stiffening, increased left ventricular (LV) filling pressures, and ultimately, diastolic heart failure. Since impaired filling is a hallmark of the disease, regardless of comorbidities, addressing the mechanical aspects of cardiac dysfunction at the level of myofilaments, provides a direct approach to improve diastolic performance across diverse HFpEF phenotypes.

Titin is a giant myofilament protein and functions as a molecular spring, which generates passive force when sarcomeres are stretched, thereby aiding in returning the sarcomere to its resting length. Titin contributes up to ∼70% of LV physiological passive stiffness.^7,8^ In HFpEF, increased titin stiffness has been identified as a key pathological factor contributing to LV diastolic dysfunction in human and animal models.^5,9,10^

In the adult heart, there are two main isoforms of titin: N2B (∼3.0 MDa), and N2BA (∼3.3 MDa); at early development, a longer foetal cardiac titin (FCT; ∼3.5 MDa or larger) is expressed.^11,12^ These isoforms vary in the lengths of titin’s I-band region and stiffness, with the N2B isoform being the stiffest and the FCT being the most compliant. The switch between isoforms occurs during developmental periods and in disease states, regulated by specific splicing factors.^13,14^

RNA binding motif-20 (RBM20) is a major splicing regulator that determines isoform expression of titin and other sarcomeric and non-sarcomeric proteins.^15–17^ Complete inhibition of Rbm20 activity leads to the expression of N2BA-G titin (∼3.9 MDa), which is very long and highly compliant. Mice expressing N2BA-G titin exhibit reduced LV chamber stiffness and attenuated systolic contractility.^11^ Meanwhile, partial inhibition of RBM20 activity results in the expression of N2BA-N titins (∼3.5 or 3.6 MDa), which are larger than the N2BA (∼3.3 MDa) but not as large as the N2BA-G isoform. Mice expressing N2BA-N titins show reduced LV chamber stiffness while maintaining normal baseline systolic function and enhanced exercise tolerance.^11,18^

*In vivo* inhibition of Rbm20 can be achieved using palmitoylated antisense oligonucleotides (ASOs) targeting the 3′ untranslated region of Rbm20 mRNA.^19^ When administered in mice, Rbm20-ASOs decrease Rbm20’s transcript and protein expression and promote the expression of N2BA-N titins in the heart. This study is the first to explored the potential therapeutic effects of Rbm20-ASOs in a clinically relevant cardiometabolic HFpEF mouse model, which was induced by a 2-hit regimen combining a high-fat diet with L-NAME in drinking water,^20^ effectively mimicking the complex comorbidities in human HFpEF.

Accordingly, Rbm20 ASOs were administered subcutaneously once a week for 6 weeks, aiming for N2BA-N titin to comprise 50% of the total titin. We assessed cardiac function using echocardiography, pressure–volume (PV) analysis, and cardiomyocyte force measurements, and performed RNA sequencing to identify associated gene expression changes. We found that targeted isoform switching to the compliant N2BA-N titins significantly improved diastolic function, even in the context of cardiometabolic stress. This study supports the potential of Rbm20-ASOs as a targeted therapeutic intervention for HFpEF.

## 2. Methods

### 2.1 Sex as a biological Variable

Our study exclusively examined male mice, as female 2-hit mice were resistant to HFpEF development.^21^

### 2.2 Study design

The objective of this study is to evaluate the effects of Rbm20-ASO treatment in HFpEF-like conditions. We hypothesize that Rbm20-ASOs effectively reduce titin stiffness and improve diastolic function in these conditions. Our study examined male mice. Adult male C57BL/6N mice were used as they can be induced to develop a HFpEF-like condition.^22^ We used the 2-hit regimen,^20^ in which mice were fed with a high-fat diet (D12492, Research Diet Inc) and L-NAME 0.5 g/L (nitric oxide synthase inhibitor) in drinking water, beginning at 3 months of age and continue for 16 weeks. Control mice were fed with a control diet (D12450K, Research Diet Inc) and water without L-NAME. After 16 weeks on the 2-hit regimen, when the mice had developed the HFpEF-like phenotype, 2hit mice and control mice were randomly received Rbm20-ASOs or PBS treatment. Rbm20-ASOs were administered through subcutaneous injections at 25 mg/kg, once a week for 6 weeks. Following this treatment period (6 weeks of ASO/PBS treatment, or 22 weeks in a 2-hit regimen), these mice were employed in endpoint studies. Mice that developed skin diseases or those in the 2-hit group that gained less than 60% of their body weight after 16 weeks on the 2-hit regimen were excluded from the study. The estimated sample sizes were calculated by a power analysis using G*Power version 3.1.9.7 based on a 2-way ANOVA (fixed effect) of preliminary and previous published data. Investigators were blinded whenever possible. Information about the number of cardiomyocytes/measurements per animal was described in figure legends.

### 2.3 Study approval

All procedures were performed according to the NIH Guide for the Care and Use of Laboratory Animal and approved by the Institutional Animal Care and University Committee of the University of Arizona. Measures were taken to minimize animal suffering, including appropriate anaesthesia and humane endpoints. Echocardiographic studies, ECG recordings, and PV analyses were performed under isoflurane anaesthesia (2–3%) in an oxygen mixture. Heart rate, respiratory rate, and toe withdrawal reflex were closely monitored to ensure the maintenance of adequate anaesthesia. Euthanasia was carried out via cervical dislocation or exsanguination under deep isoflurane anaesthesia.

*Rbm20-ASOs* were synthesized by Eurogentec, following the sequence detailed in Radke *et al.*^19^ Mice were subcutaneously injected with *Rbm20*-ASO at 25 mg/kg, once a week for a duration of 6 weeks. The control group received PBS, which serves as the solvent for ASO.

*Titin isoform analysis* was performed as previously described.^23^ Briefly, the solubilized samples were electrophoresed on 1% agarose gels using a vertical SDS-agarose gel system (Hoefer),^24^ then stained using Coomassie brilliant blue.

### 2.4 Mouse echocardiography

Mice were anesthetized under 2% isoflurane in oxygen mixture. Transthoracic echo images were obtained with a Vevo 3100 High Resolution Imaging System (Visual-Sonics). Standard imaging planes, M-mode, Doppler, and functional calculations were obtained according to American Society of Echocardiography guidelines.

### 2.5 *In-vivo* PV measurements

An *in-vivo* PV analysis was performed in mice using a SciSense Advantage Admittance Derived Volume Measurement System. Mice were anesthetized and ventilated. The catheter was inserted into the LV via apical approach. The IVC (inferior vena cava) was located and occluded during a sigh (pause) in ventilation to acquire load-independent indexes. EDPVR was analysed using a mono-exponential fit ($P=C+Ae^{\beta V}$) with the exponent (*β*) reported as the stiffness.^25^

### 2.6 Intact cardiomyocytes

Cells were isolated, as described previously.^26^ Briefly, the heart was removed and cannulated via the aorta and perfused with Liberase TM (Roche Applied Science). All intact cell experiments were performed at 37°C, and were field-stimulated at 4 Hz.

### 2.7 Loaded intact cardiomyocytes

The myocyte was attached at one end to a glass rod that connected to the force transducer. The other end of the cell was attached to a glass rod connected to the piezo translator. The cell work loop algorithm was applied as described.^27,28^ The cross-sectional area of the intact cell was obtained from the measured cell width, assuming that the cell’s cross-section was an ellipse.^7^ All forces were normalized to stress. The ED-SSLR (end-diastolic stress-sarcomere length (SL) relation) and ES-SSLR (the end-systolic stress-SL relation) were fit with linear relation.

### 2.8 Measurement of Ca^2+^ in unloaded intact cardiomyocytes

Ca^2+^ release and reuptake were measured using Fura-2 AM. The ratio of fluorescence intensities excited at 340 and 380 nm was used as a relative measurement of cytoplasmic Ca^2+^. The transient parameters were obtained from the monotonic transient analysis.

### 2.9 Permeabilized cardiomyocyte passive stiffness measurement

Cardiomyocytes were mechanically isolated from frozen LV tissue in cold relaxing solution by homogenizer, and permeabilized (skinned) in relaxing solution with 0.3% Triton X- 100. Skinned myocytes were glued to a force transducer and a servomotor. Passive stress was measured in relaxing solution (pCa 9) at 15°C. The cell was then stretched at a rate of 100% of L_O_/sec to get passive SL ranges from 1.80 to 2.50 µm. After the stretch/relaxation protocol the cell was then maximally activated to obtain the max active tension the cell could produce. To correct for ∼20% lattice expansion during skinning process,^29^ CSA of skinned cells were divided by a correction factor of 1.44. The stress was plotted against the SLs with an exponential fit to derive stress-SL relationships.

### 2.10 Picro Sirius Red for collagen quantification

Glutaraldehyde-fixed LV cross-sections were stained with Picrosirius red staining, then analysed for collagen area using image J.

### 2.11 Mouse electrocardiogram

Mice were anesthetized with oxygen and 2% isoflurane, and electrocardiogram (ECG) recordings were obtained using needle electrodes (iWorx 3-lead ECG system). An average of 200–300 beats was taken and utilized to represent one animal.

### 2.12 RNA sequencing

Sequencing libraries were prepared using the Illumina TruSeq Stranded protocol. Samples were sequenced using Illumina NovaSeq X Plus with around 100 million reads per sample and 150 bp single-end reads. First, adapters and low-quality reads were removed using fastp (v0.23.2).^30^ Remaining reads were aligned to the mouse reference genome GRCm39.110 (Ensembl) using STAR (v2.7.8a).^31^ Quality control analysis using fastqc (v0.11.9) identified a high rate of duplicated reads, which were marked and removed with Picard (v2.27) and samtools (v1.19).^32^ Differential gene expression analysis was performed using DESeq2 (v1.42.0).^33^ Genes were called differentially expressed with an adjusted *P*-value < 0.05 and an absolute log2 fold change > 0.5. Alternative splicing analysis were performed using rMATS (v4.0.2),^34^ and a splicing event was called significant with a FDR < 0.05 and an absolute delta percent spliced in (dPSI) > 0.1. Data analysis was performed in R (v4.3). The R package clusterProfiler (v4.10.0)^35^ was used for gene enrichment analyses. PSI calculation was performed using the psi_python scripts from https://github.com/MIAOKUI/PSI^36^ and a customized gene annotation (GTF) file containing only the canonical Ttn isoform (ensembl ENSMUST00000099981) information.

### 2.13 Statistics

Statistical analysis was performed in Graphpad Prism 10 (GraphPad Software, Inc). Data are shown as mean ±SD, except for *Figure 4G*, which shown as mean ± SE, for simplicity. Statistical significance was set at *P* < 0.05. * *P* ≤ 0.05 ** *P* ≤ 0.01 *** *P* ≤ 0.001 **** *P* ≤ 0.0001. Normality of data were tested with the D’Agostino and Pearson and Shapiro–Wilk tests. Homogeneity of variance was tested with Brown–Forsythe and Bartlett’s test or *F*-test. Outliers were identified using the ROUT method with a *Q*-value of 10%. For data that were normally distributed with homogeneity of variance, differences between groups were assessed by: the unpaired *t* test (for two groups); the one-way ANOVA (for 3 groups); and the two-way ANOVA (for data with two controlled variables). For data that were not normally distributed: the Mann–Whitney *U* test was used to compare 2 groups, the Kruskal–Wallis was used to compare three groups; and logarithmic transformation followed by two-way ANOVA was used to compare data with two controlled variables. Differences between groups were assessed by the two-way ANOVA with a Tukey test for multiple comparisons (for data with two controlled variables). The Spearman’s rank was used for correlation analysis (*Figure 3J* and *K*). A mono-exponential curve fit and non-linear regression analysis with a least squares fitting method were used to determine individual curve fit differences (*Figure 4G*) among the experimental groups.

## 3. Results

### 3.1 Optimization of ASO dosage to achieve 50% N2BA-N titin isoform of total titin

**Figure 1:**
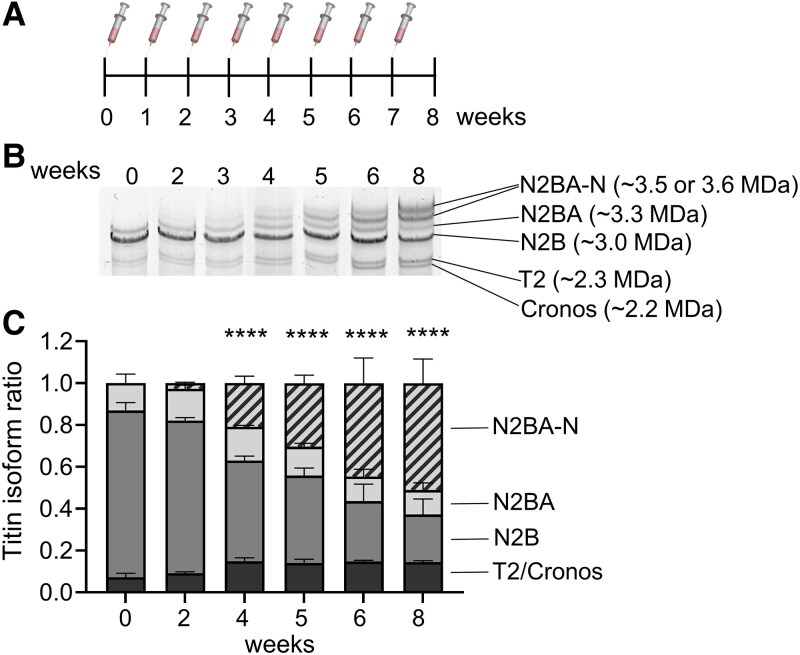
Titin isoform expression in LV myocardium of control C57BL/6n mice after weekly ASO injections. (*A*) Rbm20-ASO was administered subcutaneously at 25 mg/kg weekly to control mice. (*B*) Mice were sacrificed at the indicated time points for titin protein isoform analysis. (*C*) A significant amount of N2BA-N titins was expressed starting after 4 weeks. After six injections, 50% of the total titin is N2BA-N titin. The data are presented as means ± SD, **** *P* ≤ 0.0001 indicates a significant increase in N2BA-N compared to no injections (*n* = 7, 3, 3, 2, 2, 2 mice for weeks 0, 2, 4, 5, 6, 8, respectively)—comparison using 2-way ANOVA with Dunnett’s multiple comparisons.

Our goal was to partially inhibit RBM20 function and express N2BA-N titins to a level sufficient to achieve diastolic benefits while avoiding compromised systolic function and mitigating mis-splicing of RBM20’s additional mRNA targets. This is based on our earlier work, where 50% reduction in RBM20 led to 50% N2BA-N (supercompliant) titins and a therapeutic effect.^11^ To achieve this, we initiated a dose-finding and dose-duration study in adult male BL6N mice. ASO at 10 mg/kg led to a slow expression of N2BA-N titins that did not reach 50% by the end of week 8, while ASO at 50 mg/kg resulted in an isoform switching to N2BA-G at the end of week 8 (see Supplementary material online, *Figure S1*). We settled on a 25 mg/kg dose administered via subcutaneous injections once a week over 8 weeks (*Figure 1A*). This resulted in a progressive increase in N2BA-N titins in the LV (*Figure 1B*). While the untreated mice expressed adult wild-type N2B and N2BA titins. The Rbm20-ASOs treated mice expressed wild-type N2B and N2BA as well as N2BA-N titins. A significant quantity of N2BA-N titins was observed after 4 weeks, with the N2BA-N to total titin ratio reaching ∼0.5 after 6 weeks (*Figure 1C*). This titin analysis also confirmed successful drug delivery and suggested that the ASO dosage can be adjusted to obtain varying levels of N2BA-N.

### 3.2 Experimental design to evaluate the impact of Rbm20-ASOs in HFpEF mouse model

**Figure 2:**
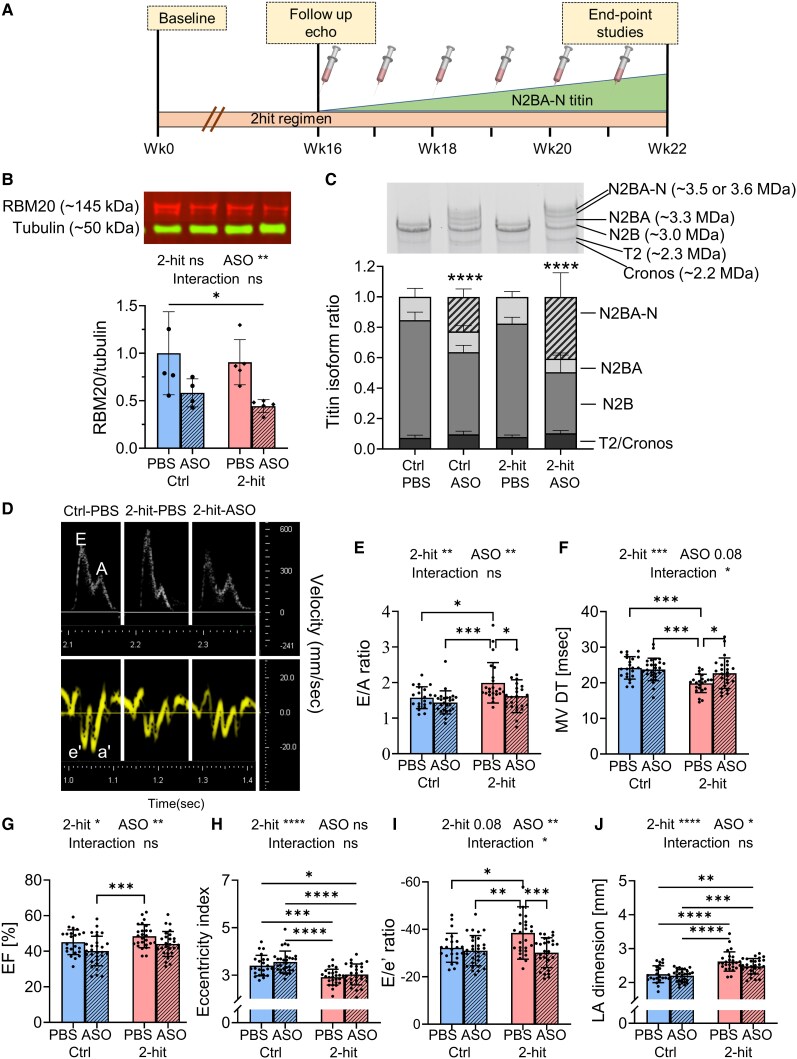
Rbm20-ASO treatment in a 2-hit model down-regulates Rbm20 expression and restores cardiac function. (*A*) Schematic of HFpEF experimental protocol. Adult male C57BL/6N mice underwent a 2-hit regimen to induce HFpEF conditions. Following this, Rbm20-ASOs were administered once a week for 6 weeks. Subsequently, these mice were employed in endpoint studies. (*B*) Western blot analysis of RBM20 with β-tubulin normalization revealed a down-regulation of RBM20 in the LVs following ASO treatment. (*C*) Titin isoform analysis indicated an up-regulation of compliant N2BA-N titins (represented by striped bars) in the ASO-treated groups. (*D*) Examples of mitral inflow pattern (upper panel) and mitral annular velocity (*D*, lower panel) from echocardiogram after 6 weeks of ASO/PBS treatment. 2-hit-PBS mice showed HFpEF-like conditions characterized by an increased *E*/*A* ratio (*E*), shortened *E* wave deceleration time (*F*), preserved LV ejection fraction (*G*), increased concentricity (*H*), elevated *E*/*e*′ ratio (*I*), and left atrial enlargement (*J*). Rbm20-ASO treatment in 2-hit mice normalized the *E*/*A* ratio (*E*), *E* deceleration time (*F*), and *E*/*e*′ ratio (*I*). The data are presented as means ± SD. * *P* ≤ 0.05, **P ≤ 0.01, ***P ≤ 0.001, ****P ≤ 0.0001, ns indicates not statistically significant. The sample sizes of Ctrl-PBS, Ctrl-ASO, 2-hit-PBS, and 2-hit-ASO were 23, 29, 26, 27 mice for echocardiogram, 5, 4, 5, 5 mice for RBM20 western blot, and 10, 10, 10, and 10 mice for titin isoform analysis. Statistical analysis was performed using 2-way ANOVA with Tukey’s multiple comparisons. Two-way ANOVA analysis results are shown above each figure. Additional echocardiographic parameters are provided in Supplementary material online, *Table S1*.

As outlined in *Figure 2A*, we divided the experimental animals into four groups: Ctrl-PBS (solvent), Ctrl-ASO, 2-hit-PBS, and 2-hit-ASO. Male C57BL/6N mice, beginning at 12 weeks of age, were subjected to a 2-hit regimen to induce cardiometabolic HFpEF.^20^ The PBS vehicle control was selected to facilitate comparison to our earlier work with the identical experimental design.^19^ Control mice were fed a control diet with normal water. After 16 weeks on the 2-hit regimen, when the mice had fully developed the HFpEF-like phenotype, Rbm20-ASOs were administered through subcutaneous injections at 25 mg/kg, once a week for 6 weeks, to achieve the N2BA-N titins to 50% of total titin as determined in the dose duration study (*Figure 1C*). Following this treatment period (6 weeks of ASO/PBS treatment, or 22 weeks in a 2-hit regimen), these mice were employed in endpoint studies.

Rbm20-ASO treatment reduces RBM20 protein expression in control and 2-hit mice, confirmed in a RBM20 western blot (*Figure 2B*, expression ratio ∼0.5, *P* < 0.01 for ASO effect). Titin isoform analysis revealed an elevation of N2BA-N titins (*Figure 2C*), corresponding with the down-regulation of the RBM20 protein (*Figure 2B*).

### 3.3 Rbm20-ASOs ameliorate LV diastolic dysfunction

#### 3.3.1 LV diastolic filling was improved in the 2-hit mice after ASO treatment

Echocardiography reveals diastolic dysfunction with preserved ejection fraction in the 2-hit-PBS mice. Two-hit-PBS showed an elevated *E*/*A* ratio (*Figure 2D* and *E*) and a shortened E wave deceleration time (*Figure 2F*), indicating a restrictive pattern of LV diastolic filling, while the ejection fraction remained preserved (*Figure 2G*). Additionally, there was a decrease in LV eccentricity (*Figure 2H*), signifying LV concentric remodelling. The *E*/*e*′, a reliable predictor of LV filling pressure, was elevated (*Figure 2I*), corresponding with the enlargement of the left atrial (LA) dimension (*Figure 2J*), indicating elevated LV filling pressure.

Rbm20-ASO treatment positively impacts these parameters, as observed in the 2-hit-ASO mice. ASO treatment normalized the *E*/*A* ratio (*Figure 2E*), *E* wave deceleration time (*Figure 2F*), and *E*/*e*′ (*Figure 2I*), suggesting an improvement in diastolic function and a reduction in LV filling pressure. None of these parameters exhibit significant differences between the Ctrl-PBS and Ctrl-ASO groups, indicating a mild effect of Rbm20-ASO in the Ctrl condition (*Figure 2E–J*). ASO treatment did not reverse LV concentric remodelling (*Figure 2H*) or cause a significant reduction in ejection fraction (*Figure 2G*) in the 2-hit mice. Thus, following ASO treatment, LV, LA, RV, and RA dimensions were not significantly different from PBS-treated controls (see Supplementary material online, *Figure S2*—normalized to tibia length). In contrast, all these cardiac compartments are significantly increased in the PBS-treated HFpEF group, indicating that the ASO treatment effectively restores cardiac structure towards normal. The detailed echocardiographic results are presented in Supplementary material online, *Table S1*.

#### 3.3.2 Improvement of LV stiffness and relaxation in 2-hit mice after ASO treatment

**Figure 3:**
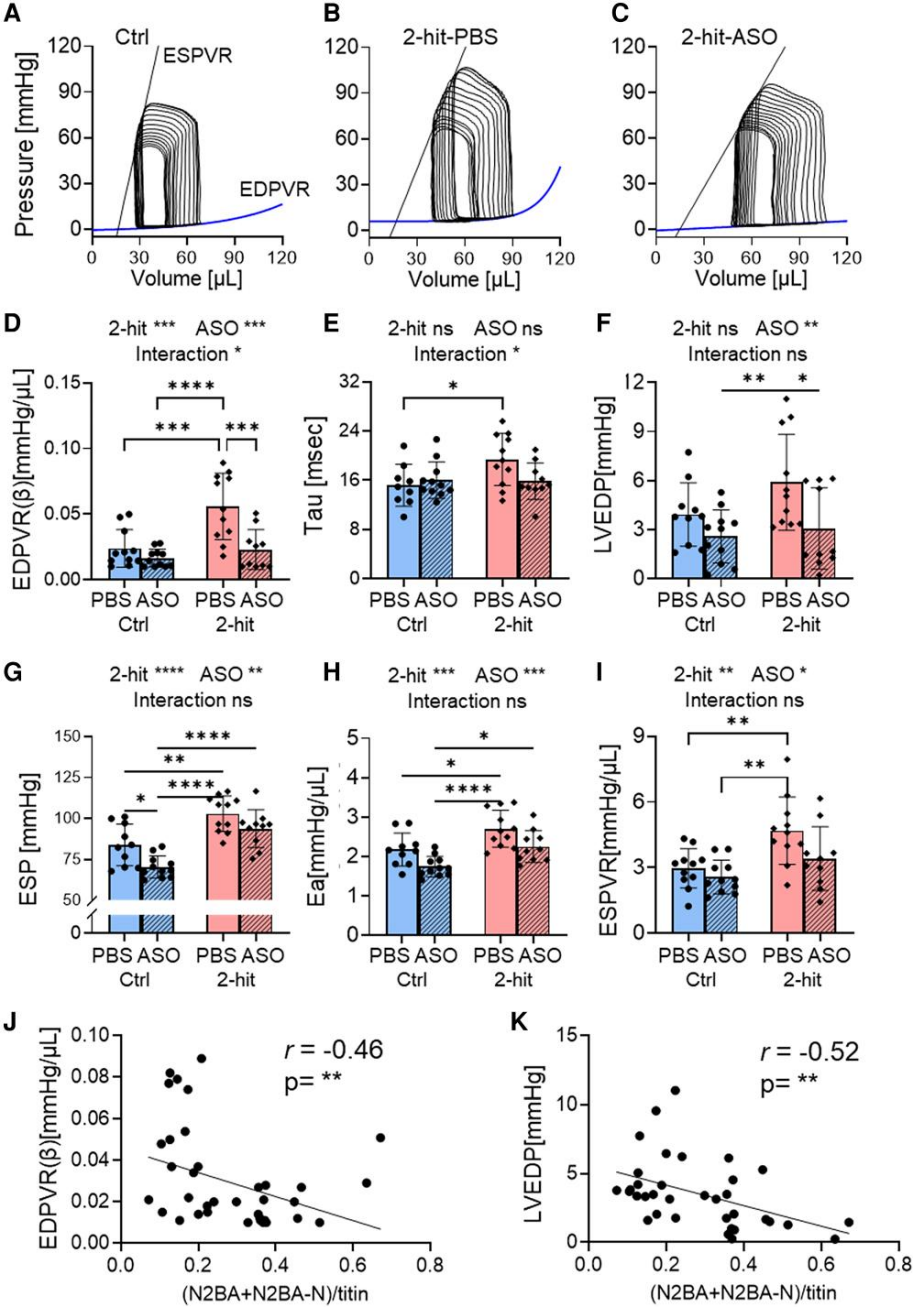
PV analysis of 2-hit mice after Rbm20-ASO treatment. Representative PV analysis of Ctrl-PBS (*A*), 2-hit-PBS (*B*), and 2-hit-ASO (*C*) mice. The 2-hit-PBS mice had increased End Diastolic Pressure–Volume Relation (EDPVR (β); *D*), increased relaxation constant (*E*), elevated LV End-Diastolic Pressure (LVEDP; *F*), heightened LV End-Systolic Pressure (LV ESP; *G*), increased Effective Arterial Elastance (Ea; *H*), and preserved End-Systolic Pressure–Volume Relation (ESPVR; *I*). Treatment with Rbm20-ASOs normalized EDPVR (β) (*D*), relaxation constant (*E*), and LVEDP (*F*). (*J*, *K*) EDPVR and LVEDP are inversely correlated with the ratio of compliant titin isoforms (N2BA and N2BA-N titins). The data are presented as means ± SD. The sample size included *n* = 11, 11, 11, and 10 mice for Ctrl-PBS, Ctrl-ASO, 2-hit-PBS, and 2-hit-ASO, respectively. * *P* ≤ 0.05, ** *P* ≤ 0.01 ***, *P* ≤ 0.001, ****P ≤ 0.0001, ns indicates not statistically significant. The analysis employed Two-way ANOVA with Tukey’s multiple comparisons and Spearman’s rank for correlation analysis. Two-way ANOVA analysis results are shown above each figure. Additional PV analysis parameters are available in Supplementary material online, *Table S2*.

The 2-hit-PBS mice exhibited parameters that are indicative of diastolic dysfunction. The diastolic stiffness coefficient (*β*) of EDPVR (end-diastolic PV relation), reflecting LV chamber diastolic stiffness (*Figure 3D*), the LV relaxation time constant (Tau) representing the time of isovolumetric LV relaxation (*Figure 3E*), and the LVEDP (LV end-diastolic pressure; *Figure 3F*) were all elevated in the 2-hit-PBS mice. Additionally, there was an increase in ESP (End-Systolic Pressure; *Figure 3G*) and Ea (Effective arterial elastance; *Figure 3H*), which were attributed to metabolic-induced systemic hypertension. The slope of ESPVR (End-systolic PV relation), indicating LV contractility, was raised in the 2-hit-PBS mice (*Figure 3I*), likely compensating for increased arterial load (higher Ea; *Figure 3H*) to maintain normal cardiac output. These findings align with the echocardiographic data, confirming diastolic dysfunction with preserved ejection fraction in the 2-hit-PBS mice.

Rbm20-ASO treatment normalized several parameters, including LV chamber stiffness (EDPVR(β); *Figure 3D*), and LVEDP (*Figure 3F*), with a decreasing trend observed in the relaxation time constant (*Figure 3E*), which normalized the time constant of 2-hit-ASO to the level observed in control mice. Unlike the 2-hit-PBS mice, the 2-hit-ASO mice did not exhibit elevated systolic contractility (ESPVR; *Figure 3I*), consistent with previous findings in the Rbm20 genetic mouse model where increased compliant titins attenuated systolic contractility.^11^ However, ventricular-arterial (VA) co-ordination, represented by the coupling ratio of Ea/ESPVR, which indicates the relationship between ventricular contractility and arterial elastance, was maintained in both groups of 2-hit mice (see Supplementary material online, *Table S2*). This suggests that despite having lower systolic contractility (ESPVR) compared to the 2-hit-PBS mice, the 2-hit-ASO mice maintain normal pumping function (VA co-ordination). Detailed hemodynamic parameters can be found in Supplementary material online, *Table S2*.

Linear regression analysis revealed negative correlations between the expression ratio of compliant titins (N2BA and N2BA-N) and LV chamber stiffness (EDPVR (β); *Figure 3J*) as well as LV EDP (*Figure 3K*). This indicates that a higher expression ratio of compliant titins (N2BA and N2BA-N) was associated with improved LV chamber stiffness (*Figure 3J*; *r* = −0.46, *P* < 0.01), and reduced LV filling pressure (*Figure 3K*; *r* = −0.52, *P* < 0.01), supporting the notion that isoform switching to compliant titins alleviates LV diastolic dysfunction.

In addition to LV function, the inhibition of Rbm20 demonstrated a mitigating effect on the hypertrophic remodelling of the left and right ventricles in the 2-hit ASO (see Supplementary material online, *Figure S2* and *Table S3*). This is likely due to the mechanosensory function of titin, which regulates muscle trophicity, where low-stiffness titin attenuates muscle hypertrophy.^37,38^ Furthermore, normalization of the LA weights in the 2-hit-ASO group (see Supplementary material online, *Figure S2*) also supports an improvement in diastolic filling observed in echo and PV analysis.

In summary, LV diastolic function was restored in the 2-hit mice after ASO treatment, documented by both Echocardiography (*E*/*E*′, *Figure 2I*) and *In-vivo* PV measurements (LVEDP, *Figure 3F*).

### 3.4 Rbm20-ASOs ameliorate diastolic dysfunction at the cellular level

**Figure 4:**
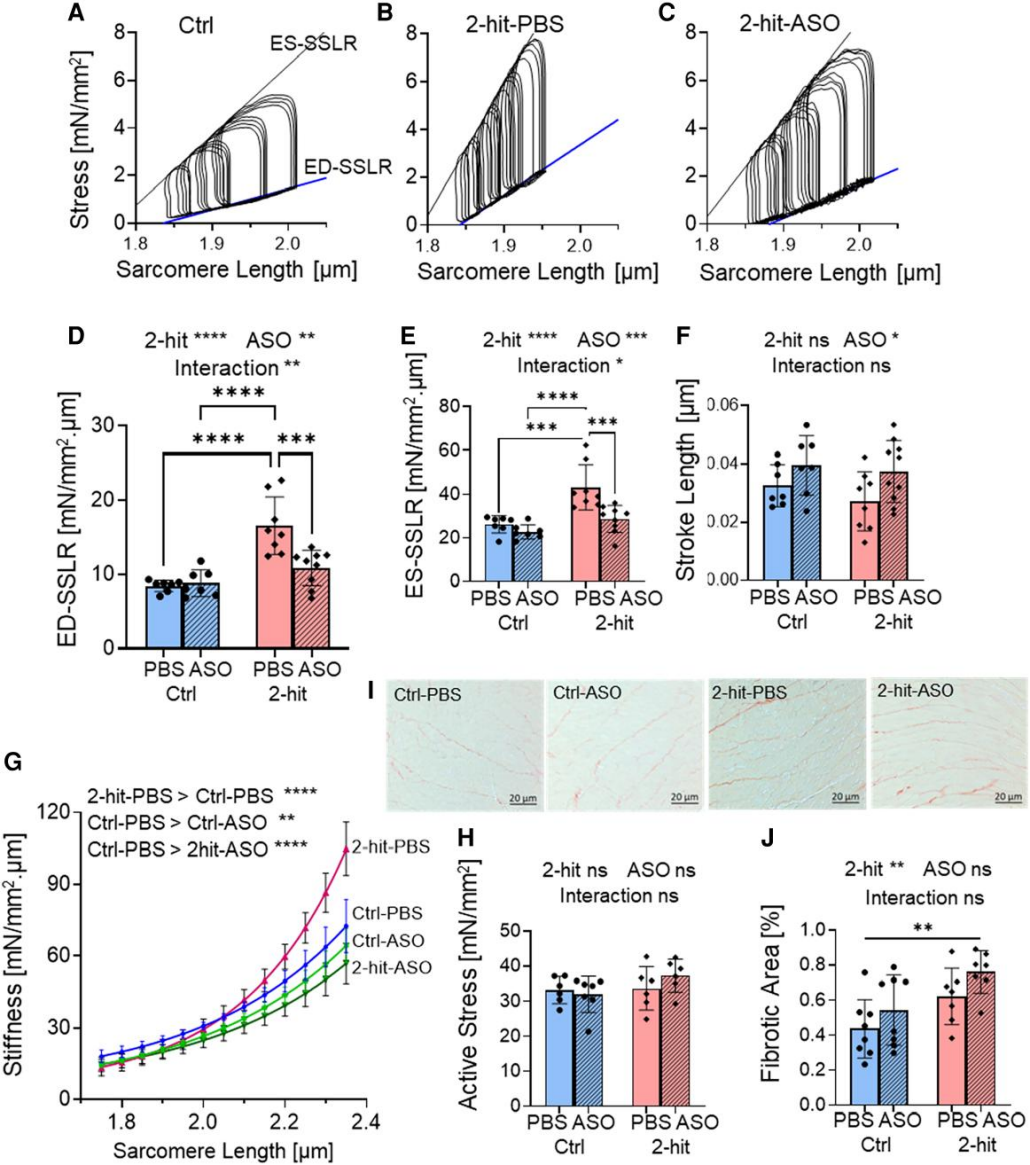
Cardiomyocyte contributions to LV diastolic stiffness. The intact cardiomyocyte stress-sarcomere length (SL) analysis is shown in (*A*-*F*). (*A*-*C*) shows examples of cellular work loops of intact cardiomyocytes from Ctrl-PBS (*A*), 2-hit-PBS (*B*), and 2-hit-ASO (*C*). Intact cardiomyocytes from 2-hit-PBS hearts demonstrate increased slopes of End-Diastolic Stress-Sarcomere Length Relation (ED-SSLR; *D*) and End-Systolic Stress-Sarcomere Length Relation (ES-SSLR; *E*). The ASO treatment normalized the ED-SSLR (*D*) and ES-SSLR (*E*) in the 2-hit mice. ASO treatment did increase stroke length (*F*; *P* < 0.05 for ASO effect). (*G*) The permeabilized cardiomyocytes’ passive stiffness is elevated in 2-hit-PBS and reduced in Ctrl-ASO and 2hit-ASO mice. (*H*) Maximal active stress is not different between groups. (*I* and *J*) Myocardial fibrosis was quantified by Picrosirius red staining of the LV section. The data are presented as means ± SD, except G, which showed mean ± SE, for simplicity. *** *P* ≤ 0.001, **** *P* ≤ 0.0001, ns indicates not statistically significant. For (*D*-*F*), the sample sizes were *n* = 7, 7, 8, and 9 mice for Ctrl-PBS, Ctrl-ASO, 2-hit-PBS, and 2-hit-ASO, respectively. Each data point represents the average value from one mouse, with 4–8 cells analysed per mouse. For (*G* and *H*), the sample sizes were *n* = 6, 7, 6, 6 mice, with 7–8 cells analysed per mouse. For (*J*), the sample sizes were *n* = 8, 8, 7, 7 mice. Two-way ANOVA with Tukey’s multiple comparisons was used for *D–F*, *H*, and *J*). Two-way ANOVA analysis results are shown above each figure. Non-linear regression analysis with a least squares fitting was performed for (*G*). Additional mechanical parameters for intact cardiomyocytes are provided in Supplementary material online, *Table S4*.

To explore the diastolic dysfunction and the ASO effect on the single-cell level without the influence of the extracellular matrix (ECM), we performed measurements on intact LV cardiomyocytes isolated from the mice after 6 weeks of ASO/PBS treatment (*Figure 4*). Experiments were performed at 37°C, under 4 Hz field-stimulation. The force measurements were conducted using a cellular work loop, analogous to PV loops at the LV chamber level.^27^ Representative cellular work loops of intact cardiomyocytes are depicted in *Figure 4A–C*. The slopes of ED-SSLR (end-diastolic stress-SL relation) and ES-SSLR (end-systolic stress-SL relation) serve as indicators of cellular diastolic stiffness and systolic contractility, respectively. Intact cardiomyocytes from the 2-hit-PBS hearts exhibited increased slopes of ED-SSLR (diastolic stiffness; *Figure 4D*) and ES-SSLR (systolic contractility; *Figure 4E*). These increased slopes correlated with the elevated EDPVR(β) and ESPVR at the LV chamber level (*Figure 3D–I*), confirming diastolic dysfunction with preserved ejection fraction phenotypes. While a prolonged relaxation constant was observed at the LV chamber level, only a trend of prolonged relaxation was noted at the cellular level (see Supplementary material online, *Table S4*; 19.7 vs. 16.9 msec for 2-hit-PBS vs. Ctrl-PBS). ASO treatment normalized cellular stiffness (ED-SSLR; *Figure 4D*) but also reduced cellular contractility (ES-SSLR; *Figure 4E*), consistent with observations at the LV chamber level, due to increased compliance of titin.^11^ The stroke length (*Figure 4F*), calculated as the difference between end-diastolic SL and end-systolic SL, and is analogous to the stroke volume at the LV level, showed a significant positive effect of ASO (*P* < 0.05). This suggests that the cardiomyocytes from ASO-treated mice operate at the wider working SL range due to compliant sarcomeres. Additional mechanical parameters of intact cardiomyocytes are in Supplementary material online, *Table S4*.

Passive force measurement was conducted in skinned (permeabilized) LV cardiomyocytes in a relaxing solution without Ca^2+^ (pCa 9.0) at 15°C to measure titin stiffness. The cardiomyocytes were stretched from baseline SL (∼1.85–1.90 µm) to a targeted SL of 2.4 µm, and passive stiffness was obtained. The result showed an increased cellular passive stiffness in 2-hit-PBS mice (*Figure 4G*), while the passive stiffness of Ctrl-ASO and 2-hit-ASO was reduced compared to Ctrl-PBS (*Figure 4G*). Although the passive cellular stiffness was reduced, no difference in the maximal active stress was observed between ASO-treated and PBS-treated mice (*Figure 4H*). Since the passive stiffness of permeabilized cardiomyocytes is mainly derived from titin,^7^ the results suggest that the reduction in LV and intact cardiomyocyte stiffness in the 2-hit-ASO is attributable to a reduction of titin stiffness.

The ECM also contributes to LV diastolic stiffness, particularly in pathological conditions. We quantified the area of LV myocardial fibrosis by Picrosirius red staining of the LV section (*Figure 4I*). Two-way ANOVA showed a significant effect of 2-hit on the percent area of myocardial fibrosis (*Figure 4J*; *P* < 0.01 for 2-hit effect), and the *post hoc* analysis was only significant between the most extreme groups with increased fibrosis in 2-hit-ASO compared to Ctrl-PBS. This may result from the interaction between titin and the ECM, as previously proposed,^11,15,39^ where a decrease in titin stiffness can lead to an increase in ECM stiffness as a compensatory response. Nevertheless, the increase in ECM-based stiffness is insufficient to counteract the therapeutic effect on titin-based stiffness, as filling was improved in the therapeutic setting of the 2-hit-ASO group (*Figure 3*).

In summary, LV diastolic dysfunction in the 2-hit mice was due to cardiomyocyte diastolic dysfunction. The restoration of diastolic function following ASO treatment was attributed to reduced titin stiffness.

### 3.5 Rbm20-ASOs alter titin and Camk2d splicing

**Figure 5:**
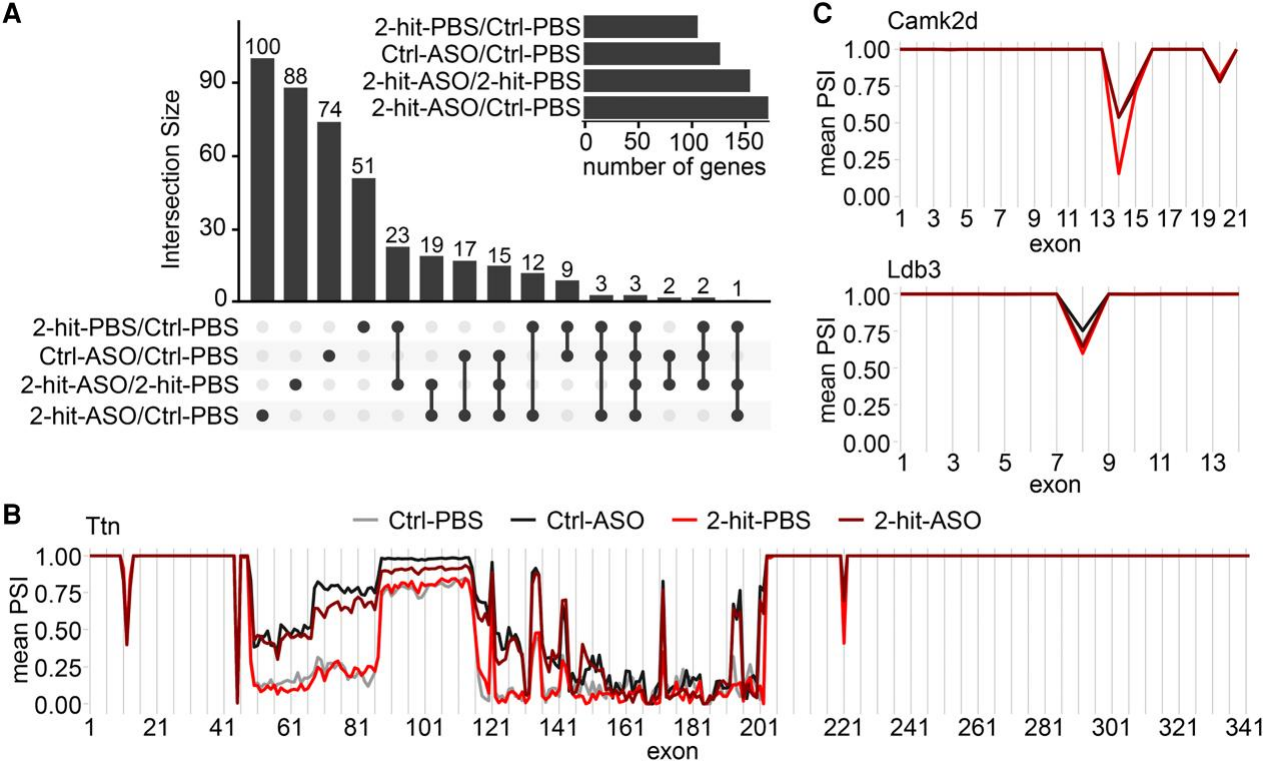
Rbm20-ASOs modify splicing of Camk2d, Ldb3, and titin. Transcriptomic analysis of LV myocardium shows differential gene splicing of Ctrl-PBS, Ctrl-ASO, 2-hit-PBS, and 2-hit-ASO after 6 weeks of ASO/PBS treatment. (*A*) Intersections of differentially spliced skipped exon (SE) events (FDR < 0.05, |dPSI| > 0.1) across the individual comparisons. (*B*, *C*) Percent spliced in (PSI) values for titin, Camk2d, and Ldb3. RNAseq, *n* = 4 mice per group.

RNA sequencing analysis was conducted to assess the effect of Rbm20-ASOs on the splicing regulation of titin and non-titin substrates, and on differential gene expression. *Figure 5A* illustrates the differentially spliced genes based on skipped exon events across the comparisons. Both the 2-hit conditions (2-hit-PBS vs. Ctrl-PBS) and ASO treatment (Ctrl-ASO vs. Ctrl-PBS) led to alternative splicing. The differential splicing observed under 2-hit conditions was likely attributable to the disease state. The genes affected by the ASO treatment in the Ctrl condition (Ctrl-ASO vs. Ctrl-PBS) differed from those affected by ASO under 2-hit conditions (2-hit-ASO vs. Ctrl-PBS); thus, the 2-hit conditions also influenced the set of genes that undergo differential splicing due to ASO treatment. This variation could stem from differences in Rbm20-associated factors or the subcellular compartmentalization of Rbm20 in physiological vs. pathological conditions. This implies that Rbm20-ASOs could impact various sets of genes depending on the subtype of HFpEF and comorbidities. Genes differentially spliced across the comparisons are displayed in Supplementary material online, *Data Files S3* and *S4*. Functional enrichment analysis of KEGG pathways, WikiPathways, and Gene Ontology (GO) categories (Biological Process, Molecular Function, and Cellular Component) did not yield any statistically significant results. However, among the differentially spliced genes in the 2-hit-ASO vs. 2-hit-PBS condition, seven genes were identified within a set of cardiac-enriched genes.^40^ Inhibition of titin splicing by Rbm20-ASO resulted in increased inclusion of titin exons (*Figure 5B*). The exon inclusions took part in the spring region, which was responsible for the mechanical properties, with no observed changes in titin’s A-band region. This finding aligned with titin gel electrophoresis, which demonstrated increased expression of N2BA-N titin isoforms, but no change in the mobility of T2 (primarily the A-band segment of titin).

In control animals, Camk2d transcripts largely excluded exon 14, which encodes the nuclear localization signal. ASO treatment increased exon 14 inclusion from <25% to >50% (*Figure 5C*). Exon inclusion in other known RBM20’s substrates was affected to a much lesser extent, including isoform expression of Ldb3, Ank3, and Ryr2 (*Figure 5C* and Supplementary material online, *Figure S3A* and *B*). Furthermore, Camk2d, Ldb3, Ank3, or RyR2 global transcript levels remained unchanged (see Supplementary material online, *Figure S3C*). The extent of titin exon inclusions and the mis-splicing of known Rbm20 substrate genes observed in this study were less pronounced than in our previous work.^19^ This may be attributed to the lower ASO dosage (25 mg/kg in this study vs. 50 mg/kg^19^) that reduced RBM20 expression to only 50% (*Figure 2B* and Supplementary material online, *Figure S3D*), thereby preserving some of its function and deviating less from the adult pattern of exon exclusion events. This also suggests that the expression levels of compliant titin can be titrated according to the severity of HFpEF conditions, and that titin and non-titin effects of ASO appear to be dose-dependent.

### 3.6 Rbm20-ASOs cause differential expression of genes related to the immune response

**Figure 6:**
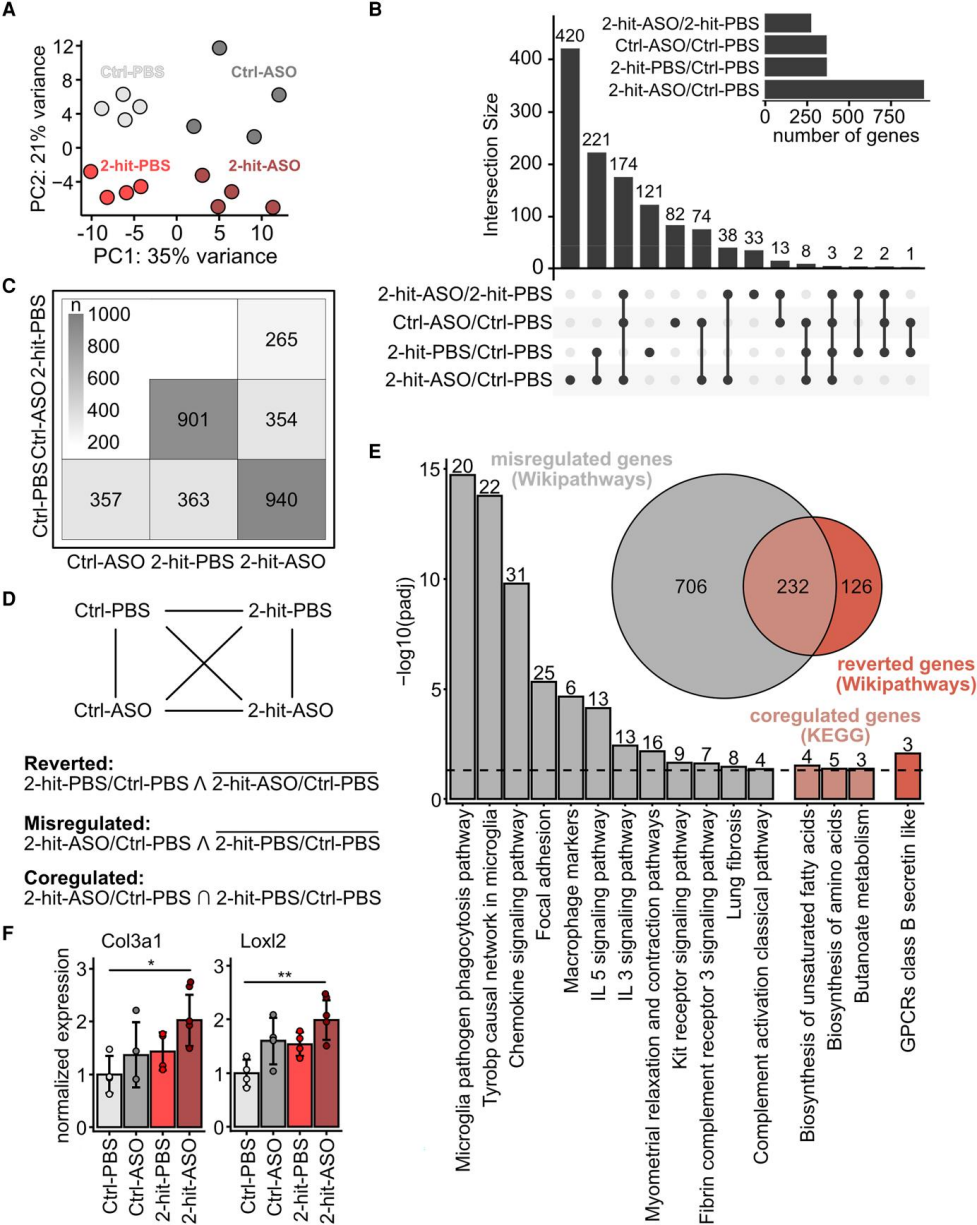
Rbm20-ASOs partially revert the differential expression caused by the 2-hit intervention and interleukin and cytokine signalling. Transcriptomic analysis of LV myocardium shows differential gene expression of Ctrl-PBS, Ctrl-ASO, 2-hit-PBS, 2-hit-ASO after 6 weeks of ASO/PBS treatment. (*A*) Principal component analysis (PCA) illustrating distinct clustering by condition. (*B*) Intersection plot showing the overlap of differentially expressed genes (adjusted *P*-value < 0.05, |log2FC| > 0.5) across the individual comparisons. (*C*) Matrix of differentially expressed genes (adjusted *P*-value < 0.05, |log2FC| > 0.5). Number of differentially expressed genes per comparison is provided in each square. The greyscale reflects the gene count as indicated by the heat map. (*D*) Genes differentially expressed between 2-hit-PBS and Ctrl-PBS but not between Ctrl-PBS and 2-hit-ASO were normalized with reduced RBM20 activity (reverted genes). Genes differentially expressed between Ctrl- PBS and 2-hit-ASO but not between Ctrl-PBS and 2-hit-PBS were additionally regulated by reduced RBM20 levels (mis-regulated genes). (*E*) Gene enrichment analysis of the mis-regulated (Wikipathways), co-regulated (KEGG), and reverted genes (Wikipathways). (*F*) Expression levels of fibrosis-associated genes, Col3a1 and Loxl2, are increased upon ASO treatment. RNAseq, *n* = 4 mice per group.

*Figure 6A–E* and Supplementary material online, *Data Files S3* (related to *Figure 6B*) and *S4* (related to *Figure 6E*) illustrate the differentially expressed genes across various comparisons. ASOs induced differential expression of genes associated with immune response (see pathway analysis in Supplementary material online, *Figure S4A*). ASO treatment reversed the differential expression of 112 genes affected by the 2-hit conditions but also led to the mis-regulation of an additional 624 genes (*Figure 6D* and *E*). Genes that were reversed, co-regulated, or mis-regulated by ASO treatment are displayed in Supplementary material online, *Data File S5*. These are secondary events as they are regulated on the level of gene expression and not on the exon level, with the latter primarily due to the effect of RBM20 on isoform expression.

We also closely assessed fibrosis-related genes. Although there was no change in the transcripts of Col3a1 and Loxl2 in the Ctrl-ASO, these transcripts were up-regulated in the 2-hit-ASO mice (*Figure 6F*), consistent with an increased collagen area fraction in histological staining of LV myocardium (*Figure 4J*). This result suggests that neither ASO nor the 2-hit intervention alone significantly increase fibrosis and that fibrosis in ASO-treated HFpEF is subtle compared to the large increase in titin compliance for a net effect of improved filling. Other fibrosis-related genes (Col1a1, Tgfb1, Timp1, and Mmp9) were not significantly altered (see Supplementary material online, *Figure S4B*). In addition, among the genes encoding for cardiac matrix-related proteins (GO:0030198), only 10 had a minor effect in splicing (see Supplementary material online, *Figure S4C*) and only two of them might relate to HFpEF via their role in pathological ECM turnover, remodelling, and fibrosis (ADAMTS6 and PTK2, alias focal adhesion kinase).^41^

### 3.7 Rbm20-ASO resulted in increased diastolic Ca^2+^ levels

**Figure 7:**
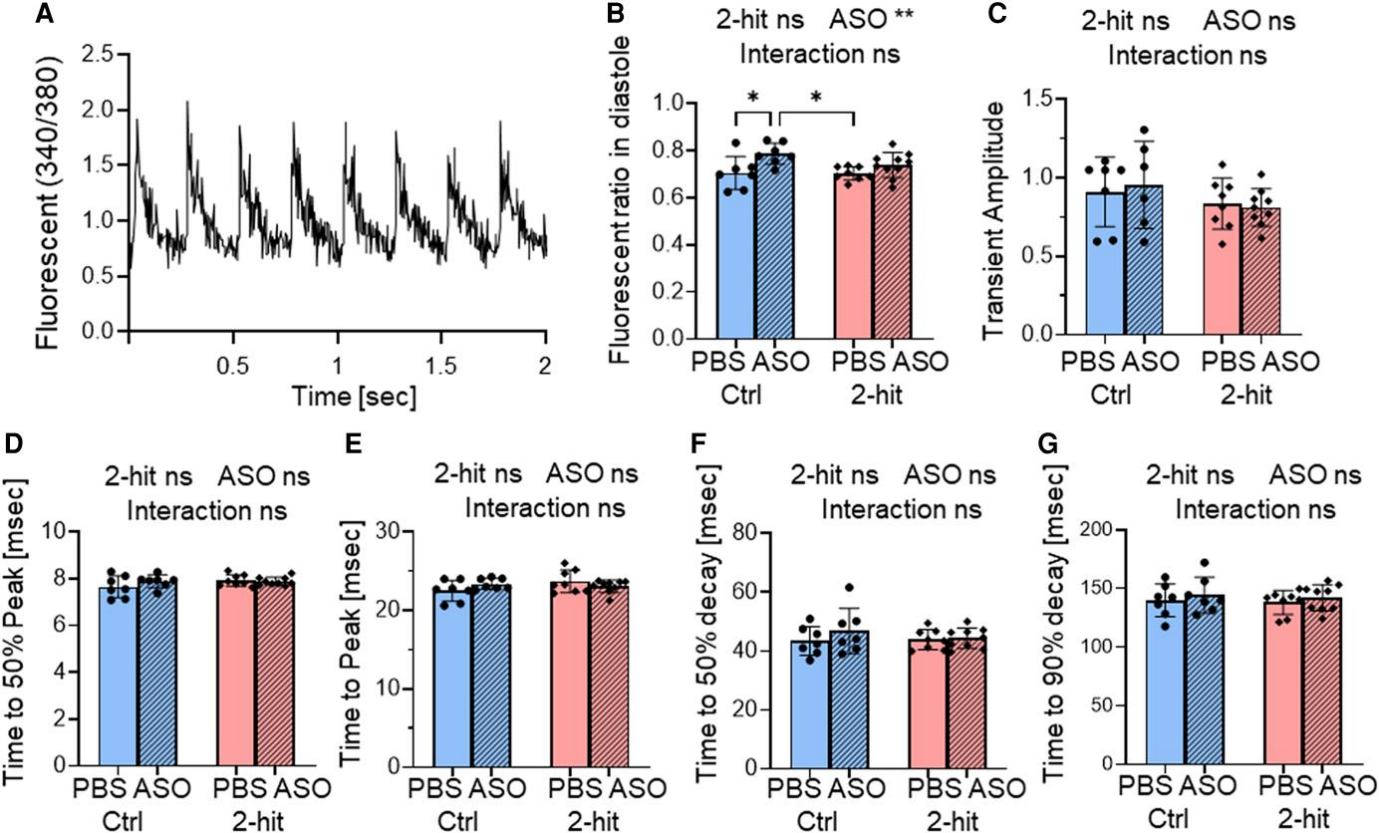
Ca^2+^ release-reuptake analysis in intact cardiomyocytes after ASO treatment. Ca^2+^ transients were measured in unloaded intact cardiomyocytes using the Fura-2 340/380 ratio at a 4 Hz stimulation rate (*A*). Diastolic cytoplasmic Ca^2+^ levels are increased in the Ctrl-ASO group (*B*). However, no significant differences are observed among groups in terms of Ca^2+^ transient amplitude (*C*), time to 50% peak (*D*), time to peak (*E*), time to 50% transient decay (*F*), and time to 90% transient decay (*G*). The data are presented as means ± SD. * *P* ≤ 0.05, ns indicates not statistically significant. Two-way ANOVA with Tukey’s multiple comparisons. Two-way ANOVA analysis results are shown above each figure. Each data point represents an average from a single animal, with 40–50 cells analysed per mouse. The study involved *n* = 7, 7, 8, and 10 mice for Ctrl-PBS, Ctrl-ASO, 2-hit-PBS, and 2-hit-ASO, respectively.

Since CaMKIIδ plays an important role in Ca^2+^ regulation within cardiomyocytes and Rbm20-ASOs modify Camk2d splicing, we analysed Ca^2+^ transients to assess the effects of ASO on Ca^2+^ release-reuptake kinetics (*Figure 7A*). Ca^2+^ transients were measured in unloaded intact cardiomyocytes using the Fura-2 340/380 ratio at 4 Hz stimulation. ASOs significantly increased diastolic Ca^2+^ levels (*P* < 0.01), specifically in the Ctrl-ASO group (*Figure 7B*). However, there was no significant effect of ASOs on Ca^2+^ transient amplitude (*Figure 7C*), Ca^2+^ release (*Figure 7D* and *E*), or Ca^2+^ reuptake kinetics (*Figure 7F* and *G*). The observed increase in diastolic Ca²⁺ in cardiomyocytes of ASO-treated mice aligns with findings from previous studies in Rbm20 homozygous genetic rats and mice.^42,43^

Considering that increased diastolic Ca^2+^ can lead to cardiac arrhythmias,^42^ we also examined the electrocardiograms (ECG) of these mice. The ECG was conducted with needle electrodes under isoflurane anaesthesia. Despite the increase in diastolic Ca^2+^ in ASO-treated mice, no signs of pro-arrhythmia were detected. There were no differences in *P* wave duration, PR interval, QRS duration, QTc interval, or T wave duration between the treated and non-treated groups (see Supplementary material online, *Figure S5A*–*F*).

## 4. Discussion

### 4.1 RBM20 inhibition and titin isoform modulation

The cardiometabolic HFpEF-like mouse model, induced by the 2-hit regimen, exhibits LV diastolic dysfunction derived from increased titin stiffness. An isoform shift towards N2BA-N titins, achieved through administration of Rbm20-ASOs, normalizes titin stiffness, reduces cellular diastolic stiffness, and restores LV diastolic function. Notably, the titin-N2BA-N isoform is similar in size to embryonic titin isoforms, although it is not identical^44^; it includes additional RBM20-dependent exons that predominantly increase the size of the elastic titin regions, thereby reducing stiffness and enhancing compliance. This beneficial effect is accompanied by a reduction in hypertrophic remodelling, and is achieved with a 50% reduced ASO dose that predominantly affects titin isoform expression and reduces side effects compared to our earlier work, thus minimizing off-target effects and improving the safety profile.^19^

In the previously published genetic mouse model, removal of titin’s elastic N2B spring element leads to diastolic dysfunction secondary to increased titin-based stiffness, which can be reverted by adding RBM20 dependent I-band exons to the mature titin protein. This approach effectively compensates for the loss of one titin spring domain by integration of other titin protein domains into the mature titin isoforms.^19^

The novelty of the current study lies in its use of a cardiometabolic HFpEF model, where systemic inflammation and metabolic disturbances drive disease progression, with multiple factors, including but not limited to titin, contributing to HFpEF development. This highlights the broader and more clinically relevant therapeutic potential of Rbm20-ASOs in treating HFpEF, particularly in the presence of complex comorbidities, offering a significant step towards addressing the multi-factorial nature of the disease.

### 4.2 Implications of RBM20 inhibition beyond titin

In addition to modulating titin, RBM20 also regulates a wide range of other genes, including Camk2d, Ldb3, Ank3, and Ryr2, which play roles in sarcomeric structure and Ca^2+^ homeostasis.^15^ Recent studies using long-read RNA sequencing technology, which provides better resolution for transcriptomic comparison than short-read RNA sequencing, revealed 107 cardiac genes with differentially expressed isoforms in the presence of an Rbm20 mutation.^45^ These genes are associated with striated muscle development, regulation of cardiac contraction, ion transport, and actin filament-based processes. Notably, one mis-spliced gene is the inner membrane mitochondrial protein (IMMT), a crucial component of the mitochondrial cristae that organizes complexes essential for mitochondrial function and metabolism.^45^

Proteomic analysis in Rbm20 knockout (KO) rats at 3 weeks of age—before the development of the DCM phenotype—revealed differential expression of 103 proteins in the Rbm20 KO.^46^ Many of these proteins are involved in mitochondrial function and metabolism, including the down-regulated enoyl-CoA hydratase 1 (ECH1), pyruvate dehydrogenase kinase 4 (PDK4), aldehyde dehydrogenase 1 family member B1 (ALDH1B1), and 3-hydroxy-3-methylglutaryl-CoA synthase2 (HMGCS2), with Hmgcs2 being the most significantly down-regulated. HMGCS2 is a critical mitochondrial enzyme that catalyses the rate-limiting step in ketogenesis and may affect the heart’s ability to efficiently manage metabolic stress.^46,47^ Additionally, cardiac proteomics of Rbm20 KO rats revealed the up-regulation of Ankrd1, which promotes hypertrophic gene expression,^48,49^ and the down-regulation of FHL2, a negative regulator of cardiac hypertrophy.^46,50^ The combined effect of increased Ankrd1 and decreased FHL2 expression could contribute to the development of hypertrophic remodelling. However, we observed attenuated hypertrophic remodelling with ASO treatment in this study (see Supplementary material online, *Figure S2* and *Table S3*), which could be attributed to other trophic signalling pathways that may override the signalling of dysregulated Rbm20-substrates.

Here, ASO treatment in the 2-hit HFpEF mouse model led to reverted gene expression of de-regulated genes that align with critical pathways implicated in HFpEF pathology. These include metabolic regulation (CPT2 and DECR1), the response to oxidative stress (catalase), inflammation and remodelling pathways (SMAD7, PYCCARD, and RETNLG), as well as endothelial function (ACE2 and FOLR2). The latter can contribute to vascular rarefication, tone, and thus cardiac workload. Normalized expression of these genes after applying a splice therapeutic ASO, suggests that they may contribute to therapeutic effects by enhancing energy metabolism and mitochondrial function, reducing oxidative stress and inflammation, adapting cardiac remodelling, and improving endothelial function and vascular health.

Additional changes involve key metabolic pathways, including fatty acid synthesis and ceramide metabolism, and xenobiotic processing (Acaca, Cers6, Aox1). Their altered splicing can modulate myocardial substrate utilization and energy homeostasis and can contribute to oxidative stress, which in turn influences ECM remodelling and overall tissue compliance. Specifically, Cers6 is involved in the synthesis of ceramides, which have been linked to HFpEF.^51^

Splicing changes also affect diverse signalling pathways via Map2k5, Tnk2, Camk2d, Erbb2, Mark2, and Rapgef6, which regulate cardiomyocyte contractility, stress responses, and adaptive remodelling, which are crucial for maintaining cardiac performance under mechanical and metabolic stress. Of these only Camk2D has been linked to diastolic dysfunction via titin phosphorylation.^52,53^

Altered splicing of transcription regulators—including Fubp3, Nfib, Zscan22, Brd4, and Med12L—and structural proteins such as Ttn, Ldb3, and Ank3 may reprogram gene expression networks that govern sarcomeric organization and ECM deposition. These changes contribute to reduced myocardial stiffness and improved diastolic function by ensuring optimal structural and functional integrity of the heart. For the sarcomeric proteins, this is well established.^54^ Among the altered transcription factors, only Brd4 has been linked to diastolic function via its role as a chromatin reader in fibroblast activation that contributes to the therapeutic effect of HDAC inhibition in HFpEF.^55^

Thus, the co-ordinated alternative splicing of this comprehensive set of genes establishes a multi-faceted regulatory network that fine-tunes metabolic processes, signal transduction, gene expression, and structural organization. This integrative effect likely underpins the enhanced myocardial compliance and diastolic performance observed following Rbm20-ASO treatment.

Taken together, RNA sequencing in our study indicates that the splicing alterations affect a wider range of genes as compared to the titin N2B KO diastolic dysfunction model. This is likely due to the influence of the systemic disease environment on RBM20 activity. This suggests that RBM20-ASOs may exert more complex regulatory effects in HFpEF associated with comorbidities, offering a potential therapeutic advantage for treating diverse HFpEF phenotypes.

Finally, we observed an up-regulation of immune response pathways following palmitoylated ASO treatment, as demonstrated by the mis-regulated genes highlighted in *Figure 6E* (Wikipathway analysis) and the increased spleen weight in treated mice (see Supplementary material online, *Table S3*). This immune activation was not observed in Rbm20 deletion animal models, suggesting it is a consequence of ASO administration rather than RBM20 inhibition itself. Therefore, it is crucial to consider immune-related side effects when developing and optimizing ASO delivery and exploring alternatives to palmitoylation.

In summary, complete inhibition of Rbm20 could lead to harmful side effects. Thus, precise titration is essential to maximize the beneficial effects on titin while minimizing unintended impact on non-titin substrates.

### 4.3 Rbm20-ASO efficacy in larger mammals and skeletal muscles

Although increased N2BA-N titin significantly reduces cardiomyocyte stiffness in the mouse model, it is important to note that this effect may not be as pronounced in humans due to differences in the proportion of adult titin isoforms among species. The ratio of cardiac N2B to N2BA titin is approximately 80:20 in mice and 65:35 in humans.^5^ Because N2BA titin is more compliant than N2B titin, the reduction in titin stiffness after Rbm20-ASO treatment is likely less pronounced in humans than in mice. Therefore, follow-up studies are warranted to test the effect of ASOs in larger mammals with titin isoform compositions closer to that of humans.

The effect of Rbm20 inhibition on mechanical function in skeletal muscles is likely minimal. Our genetic model studies indicate that titin isoforms in skeletal muscles (N2A titin) are much larger than the cardiac isoforms (N2B and N2BA titins), and the changes in titin size in skeletal muscles after Rbm20 inhibition are relatively minor^37,56^ (changes from ∼3.7 MDa to ∼3.9 MDa) compared to in the heart. There is no significant functional change in skeletal muscle function at baseline,^11,56^ suggesting that the effect of Rbm20-ASOs on skeletal muscles is likely tolerable.

### 4.4 Titin as the therapeutic target for modulating cardiac mechanical dysfunction in HFpEF

To date, clinical trials have demonstrated only two classes of drugs that improve clinical outcomes in HFpEF patients: sodium–glucose co-transporter 2 inhibitors (SGLT2i) and Glucagon-like peptide-1 receptor agonists (GLP-1RAs). SGLT2 inhibitors are glucose-lowering drugs that benefit both Heart Failure with Reduced Ejection Fraction (HFrEF) and HFpEF, partly through their anti-inflammatory activity via the inactivation of the NLRP3 (NOD-like receptor family pyrin domain-containing 3) inflammasome.^57,58^ GLP-1RAs stimulate insulin release, reduce appetite, and promote weight loss, providing particular benefits for patients with HFpEF related to obesity.^57–60^ In parallel, the strategy to target sarcomeric proteins, the downstream effectors that determine the mechanical properties of the heart potentially offers an alternative method to restore cardiac output and prevent further organ damage in a wide range of HFpEF patients, regardless of comorbidities and even in the presence of unresolved systemic comorbidities.

ASOs have already been proven to be an effective therapeutic modality in clinical settings.^61,62^ They are hydrolysed by endonucleases or exonucleases present in plasma or tissues and degradation does not heavily rely on hepatic enzymes, making ASOs particularly beneficial for use in HFpEF patients, who often present with multi-organ dysfunction.^61,62^ Combining Rbm20-ASO treatment with medications that address metabolic comorbidities may provide a comprehensive treatment for HFpEF.

### 4.5 Potential therapeutic applications of Rbm20-ASOs in other cardiac conditions

In addition to regulating diastolic function, titin acts as a mechanosensor that influences systolic contractility. Titin stiffness also enhances myofilament Ca^2+^ sensitivity and systolic force production.^11,63,64^ Consequently, reducing titin stiffness using Rbm20-ASO could benefit certain cases of hypertrophic cardiomyopathy (HCM) associated with diastolic dysfunction, hypercontractility, and LV outflow tract obstruction.^65^ Moreover, by reducing mutant RBM20 production, ASOs may mitigate the severity of Rbm20 cardiomyopathy by decreasing cytoplasmic RBM20 granule accumulation, although further investigation is required.

### 4.6 Limitations and future directions

One limitation of our study is the use of a mouse model, which may not fully replicate the human conditions. The combination of L-NAME and high-fat diet furthermore induced only a mild phenotype (i.e. only 3 mice had an LVEDP >10 mmHg). The efficacy of Rbm20-ASOs in larger animal models with titin isoform compositions closer to humans needs to be investigated. Additionally, the long-term effects and safety of Rbm20-ASOs need further investigation. This includes the prolonged expression of titin-N2BA-N isoforms, which might inadvertently impair systolic performance or activate compensatory pathways that lead to adverse remodelling. Including female subjects is very important for HFpEF therapeutic studies, since females are majority of HFpEF patients. However, we did not include female mice in this work, as female 2-hit mice were resistant to HFpEF development.^21^ Nevertheless, based on our genetic mouse model, a shift to the compliant N2BA-N titins will likely be effective in restoring diastolic function in females as well,^11^ although the pharmacokinetics and gene alterations resulting from ASO treatment may exhibit sex-dependent differences. Finally, clinical trials will be necessary to establish the therapeutic potential of Rbm20-ASOs in HFpEF patients.

## Supplementary Material

cvaf171_Supplementary_Data

## Data Availability

Detailed methods, Supplementary material online, *Figure S1–S5*, *Table S1–S4*, and *Data Files S1–S5*, Supporting data value, and R scripts for gene expression and splicing analysis are available on github (https://github.com/MitchGotthardt/GotthardtLab/tree/main/Methawasin_Granzier_Gotthardt_CVR). RNA sequencing data have been deposited to ArrayExpress and are available under the accession number E-MTAB-14882. All other data have been deposited into the Harvard Dataverse Research Data Repository under the project name ‘Rbm20 antisense oligonucleotides alleviate diastolic dysfunction in a mouse model of cardiometabolic heart failure (HFpEF)’ (https://dataverse.harvard.edu/dataverse/rbm20asoin2hitmice).
