# Supplementary material for: Rbm20 antisense oligonucleotides alleviate diastolic dysfunction in a mouse model of cardiometabolic heart failure (HFpEF)
Source: Cardiovasc Res. 2025 Oct 17;121(13):2027–41. doi: 10.1093/cvr/cvaf171 (PMC12560776; doi:10.1093/cvr/cvaf171)
Supplement: cvaf171_Supplementary_Data [file cvaf171_supplementary_data.zip › Manuscript Rbm20 ASO HFpEF r3 suppl.docx]

**SUPPLEMENTARY MATERIALS**

**Supplementary Methods**

*Experimental animals.* Adult male C57BL/6N mice were used as they can be induced to develop the full HFpEF-like condition ^1^.We used the 2-hit regimen ^2^, in which mice were fed with a high-fat diet (D12492, Research Diet Inc) and L-NAME 0.5g/L (nitric oxide synthase inhibitor) in drinking water, beginning at 3 months of age and continue for 4 months. Control mice were fed with a control diet (D12450K, Research Diet Inc) and water without L-NAME. All procedures were performed according to the NIH Guide for the Care and Use of Laboratory Animal and approved by the Institutional Animal Care and University Committee of the University of Arizona. Measures were taken to minimize animal suffering, including appropriate anesthesia and humane endpoints.

*Rbm20-ASOs* were synthesized by Eurogentec, following the sequence detailed in Radke et al ^3^. Mice were subcutaneously injected with *Rbm20*-ASO at 25 mg/kg, once a week for a duration of 6 weeks. The control group received PBS, which serves as the solvent for ASO.

*Titin isoform analysis* was performed as previously described ^4^. Briefly, the solubilized samples were electrophoresed on 1% agarose gels using a vertical SDS-agarose gel system (Hoefer) ^5^. Gels were run at 15 mA per gel for 3 h and 20 min, then stained using Coomassie brilliant blue, scanned using a commercial scanner and analyzed with One-D scan (Scanalytics Inc).

*RBM20 Western blot.* RBM20 expression levels were quantified with Western blotting as previously described ^6^. Solubilized samples were run on a 10% SDS-PAGE in a vertical gel electrophoresis chamber, then transferred onto PVDF membranes. Blots were stained with Ponceau S to visualize the total protein transferred. Blots were then probed with primary antibodies followed with secondary antibodies conjugated with fluorescent dyes. Blots were scanned using an Odyssey Infrared Imaging System (Li-COR Biosciences) and the images were analyzed using Li-COR software. Ponceau S scans were analyzed in One-D scan to normalize WB signal to protein loading.

*Mouse Echocardiography.* Mice were anesthetized under 2% isoflurane in oxygen mixture. Transthoracic echo images were obtained with a Vevo 3100 High Resolution Imaging System (Visual-Sonics) using the model MX550S scan head designed for murine cardiac imaging. Body temperature was maintained at 37°C. Standard imaging planes, M-mode, Doppler, and functional calculations were obtained according to American Society of Echocardiography guidelines. The parasternal long axis view and mid wall cross sectional view of the left ventricle (LV) were used to guide calculations of percentage fractional shortening, percentage ejection fraction, and ventricular dimensions and volumes. In addition, the left atrial dimension was measured in the long-axis view directly below the aortic valve leaflets. Passive LV filling peak velocity, E (cm/sec), and atrial contraction flow peak velocity, A (cm/sec), were acquired from the images of mitral valve Doppler flow from tilted parasternal long axis views.

*In-vivo pressure-volume measurements.* An in-vivo pressure-volume analysis was performed in mice using a SciSense Advantage Admittance Derived Volume Measurement System and 1.2F catheters with 4.5 mm electrode spacing (SciSense, London, Ontario, Canada). Mice were anesthetized and ventilated with 2% isoflurane using an SAR-1000 Ventilator (CWE Inc) and body temperature maintained at 37^o^C using a Mouse Monitor S platform (Indus Instruments). Anesthetized mice were secured, and a bilateral subcostal incision was made. The diaphragm was opened to expose the heart. The catheter was inserted into the LV via apical approach. The IVC (inferior vena cava) was located and occluded during a sigh (pause) in ventilation to acquire load-independent indexes. Data acquisition and analysis was performed in LabScribe23 (iWorx, Dover NH). EDPVR was analyzed using a mono-exponential fit ($P=C+Ae^{\beta V}$) with the exponent (β) reported as the stiffness ^7^.

*Intact cardiomyocytes.* Cells were isolated, as described previously ^8^. Briefly, mice were heparinized (1,000 U/kg, i.p.) and euthanized using isoflurane. The heart was removed and cannulated via the aorta with a blunted 21-gauge needle for antegrade coronary perfusion. The heart was perfused with perfusion buffer ([in mmol/L] 90 NaCl, 34.7 KCl, 0.6 KH_2_PO_4_,0.6 Na_2_HPO_4_, 1.2 MgSO_4_, 12 NaHCO_3_, 10 KHCO_3_, 10 HEPES, 10 taurine, 5.5 glucose, 5 BDM,20 Creatine, pH 7.4), followed by 0.05 mg/ml Liberase TM (Roche Applied Science). All intact cell experiments were performed at 37°C in Medium199 plus 10 μg/mL insulin.

*Loaded intact cardiomyocytes.* An inverted microscope (IX-70; Olympus) was used with a chamber with platinum electrodes to electrically stimulate cells, and a perfusion line with heater control and suction out to maintain a ∼2 ml/min flow rate. Cells were field-stimulated at 4 Hz by MyoPacer stimulator (IonOptix LLC). All images were recorded with a 40X objective lens. Data were collected using an IonOptix FSI A/D board and IonWizard 6.4 software (IonOptix LLC). A cellular work loop was conducted as described^9, 10^. The glass rods coated with myotak (IonOptix Co) were carefully lowered onto opposite ends of the cell. The myocyte was attached at one end to a glass rod that connected to the force transducer (OFT200, OptiForce transducer, IonOptix LLC). The other end of the cell was attached to a glass rod connected to the piezo translator (Mad City Lab). The cell work loop algorithm was applied through the interface box of the IonOptix system, which contains a field-programmable gate array (FPGA), and the preload and afterload value programmed through the IonWizard software 6.4, as described ^9^. The piezo translator adjusted cell length through a feedback control system based on developed force, implementing preload and afterload. The cross-sectional area of the intact cell was obtained from the measured cell width, assuming that the cell’s cross-section was an ellipse ^11^. All forces were normalized to stress. Data analysis was performed in LabScribe23 (iWorx, Dover, NH). The ED-SSLR (end-diastolic stress-sarcomere length relation) and ES-SSLR (the end-systolic stress-sarcomere length relation) were fit with linear relation.

*Measurement of Ca^2+^ in unloaded intact cardiomyocytes.* Isolated LV cardiac myocytes were incubated with Fura‐2 AM 2 μM (F‐1225, Life Technologies) for 10 min at room temperature and resuspended in Medium199. Intact cell experiments were performed at 37°C in Medium199 plus 10 μg/mL insulin. Cells were field-stimulated at 4 Hz. Fura‐2 was excited alternately at 340 and 380 nm, and emission was recorded at 510 nm. Background fluorescence was subtracted for each excitation wavelength. The ratio of fluorescence intensities excited at 340 nm and 380 nm was used as a relative measurement of cytoplasmic Ca^2+^. The Ca^2+^ transients were recorded by the IonOptix photometry MultiCell High Throughput system (IonOptix LLC). The transient parameters were obtained from the monotonic transient analysis.

*Skinned cardiomyocyte passive stiffness measurement.* Mouse LV tissues were flash-frozen and stored at -80 °C. Cardiomyocytes were mechanically isolated from frozen LV tissue in cold relaxing solution by Bio-Gen PRO200 homogenizer. Cardiomyocytes isolated as explained above, were skinned for 10 min in relaxing solution with protease inhibitors ([in mmol/L] 0.4 leupeptin, 0.1 E64, and 0.5 PMSF) and 0.3% Triton X‐ 100. Cells were washed extensively with relaxing solution pCa 9 and stored on ice. Myocyte suspension was added to a temperature regulated chamber mounted on the stage of an inverted microscope. Skinned myocytes were glued using aquarium sealant to a force transducer (Model 406A, Aurora Scientific) and a servomotor (Model 315C-I, Aurora Scientific) that imposes controlled stretches. Sarcomere length (SL) was measured with a Video sarcomere length software (VSL 900B, Aurora Scientific) attached to a computer. Passive stress was measured in relaxing solution (pCa 9) at 15 °C. The cell was stretched to just taut, an SL of 1.75-1.85 microns based on the FFT of the video signal captured from the cell. The cell was then stretched at a rate of 100% of L_O_/sec to get passive SL ranges from 1.80-2.50 microns. Each stretch of the cell was held for 10 seconds to allow for relaxation to occur before the cell length was shortened in a step wise manner. Each cell underwent a minimum of 4 sets of 5 stretches to sample the entire working range of the working heart of a mouse ^12^. After the stretch/relaxation protocol the cell was then maximally activated to obtain the max active tension the cell could produce. The cross-sectional area of the cell was obtained from the measured cell width and thickness, assuming that the cell’s cross-section was an ellipse ^11^. Data were collected using a real-time muscle data acquisition and analysis software (600A, Aurora Scientific) at a sample rate of 2 kHz. Measured forces were converted to stress (force/unit undeformed CSA). To correct for ~20% lattice expansion during skinning process^13^, CSA of skinned cells were divided by a correction factor of 1.44. The stress was plotted against the sarcomere lengths with an exponential fit to derive stress‐ SL relationships.

*Picro Sirius Red for collagen quantification.* Histology with Picrosirius red staining (Picro Sirius Red stain kit, abcam ab 150681) measured the collagen volume fraction in LV cross-sections. The collagen volume fraction (CVF) was measured on tissues fixed in glutaraldehyde. These fixed hearts were sliced radially into sections, embedded, sectioned, and stained using Picrosirius Red to quantify collagen content. Stained sections were then imaged on a Zeiss microscope (Imager.M1) and analyzed for collagen area using image J.

*Mouse electrocardiogram (ECG).* The ECG analysis utilized the iWorx 3-lead ECG system and was analyzed using LabScribe V23. Mice were anesthetized with oxygen and 2% isoflurane, and ECG recordings were obtained using needle electrodes. An average of 200-300 beats was taken and utilized to represent one animal.

*RNA sequencing.* Sequencing libraries were prepared using the Illumina TruSeq Stranded protocol. Samples were sequenced using Illumina NovaSeq X Plus with around 100 million reads per sample and 150 bp single-end reads. First, adapters and low-quality reads were removed using fastp (v0.23.2) ^14^. Remaining reads were aligned to the mouse reference genome GRCm39.110 (Ensembl) using STAR (v2.7.8a) ^15^. Quality control analysis using fastqc (v0.11.9) identified a high rate of duplicated reads, which were marked and removed with Picard (v2.27) and samtools (v1.19) ^16^. Differential gene expression analysis was performed using DESeq2 (v1.42.0) ^17^. Genes were called differentially expressed with an adjusted p-value < 0.05 and an absolute log2 fold change > 0.5. Alternative splicing analysis were performed using rMATS (v4.0.2) ^18^ , and a splicing event was called significant with an FDR < 0.05 and an absolute delta percent spliced in (dPSI) > 0.1. Data analysis was performed in R (v4.3). The R package clusterProfiler (v4.10.0) ^19^ was used for gene enrichment analyses. PSI calculation was performed using the psi_python scripts from https://github.com/MIAOKUI/PSI ^20^ and a customized gene annotation (GTF) file containing only the canonical Ttn isoform (ensembl ENSMUST00000099981) information.

*Statistics.* Statistical analysis was performed in Graphpad Prism 10 (GraphPad Software, Inc). Data are shown as mean ± SEM. Statistical significance was set at p<0.05. * p≤0.05 ** p≤0.01 ***p≤0.001 ****p≤0.0001. The estimated sample sizes were calculated by a power analysis using G*Power version 3.1.9.7 based on a 2-way ANOVA (fixed effect) of preliminary and previous published data. Normality of data was tested with the D’Agostino & Pearson and Shapiro-Wilk tests. Homogeneity of variance was tested with Brown-Forsythe and Bartlett’s test or F-test. Outliers were identified using the ROUT method with a Q-value of 10%. For data that were normally distributed with homogeneity of variance, differences between groups were assessed by: the unpaired t test (for 2 groups); the one-way ANOVA (for 3 groups); and the two-way ANOVA (for data with 2 controlled variables). For data that were not normally distributed: the Mann-Whitney U test was used to compare 2 groups, the Kruskal–Wallis was used to compare 3 groups; and logarithmic transformation followed by two-way ANOVA was used to compare data with 2 controlled variables. Differences between groups were assessed by the two-way ANOVA with Dunnett’s or Tukey tests for multiple comparisons (for data with 2 controlled variables). The Spearman’s rank was used for correlation analysis (Figures 3K-L). A mono-exponential curve fit and nonlinear regression analysis with a least square fitting method were used to determine individual curve fit differences (Figure 4H) among the experimental groups.

**Supplementary Figures**

N2BA-N

(~3.5 or 3.6 MDa)

N2B (~3.0 MDa)

N2BA-G (~3.9 MDa)


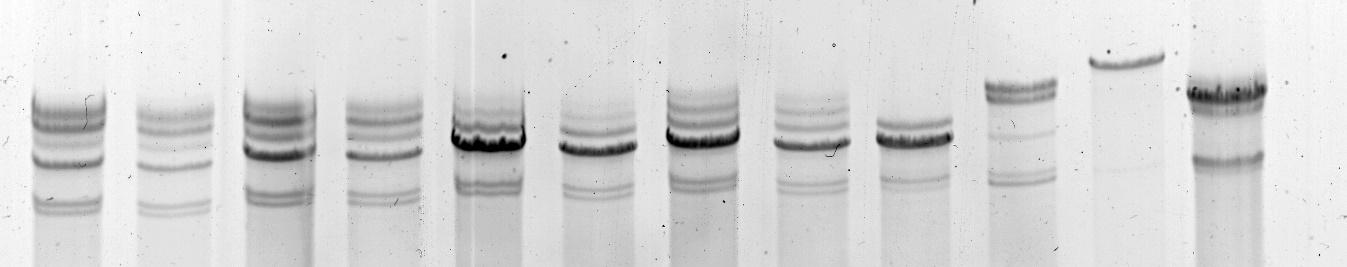


ASO

25 mg/kg

ASO

25 mg/kg

ASO

10 mg/kg

ASO

10 mg/kg

Loading

volume (µL)

4.5 9

-/- +/- WT

Rbm20

4 4 4

ASO

50 mg/kg

9 9 9

Loading

volume (µL)

9 9 9

PBS

WT -/- +/-

Rbm20

4 4 4

N2BA (~3.3 MDa)

N2B (~3.0 MDa)

T2 (~2.3 MDa)

N2BA-G (~ 3.9MDa)

N2BA-N (~3.5 or 3.6 MDa)

N2BA (~3.3 MDa)

T2 (~2.3 MDa)

4.5 9

4.5 9

4.5 9

N2B (~3.0 MDa)

Cronos (~2.2 MDa)

Cronos (~2.2 MDa)

*
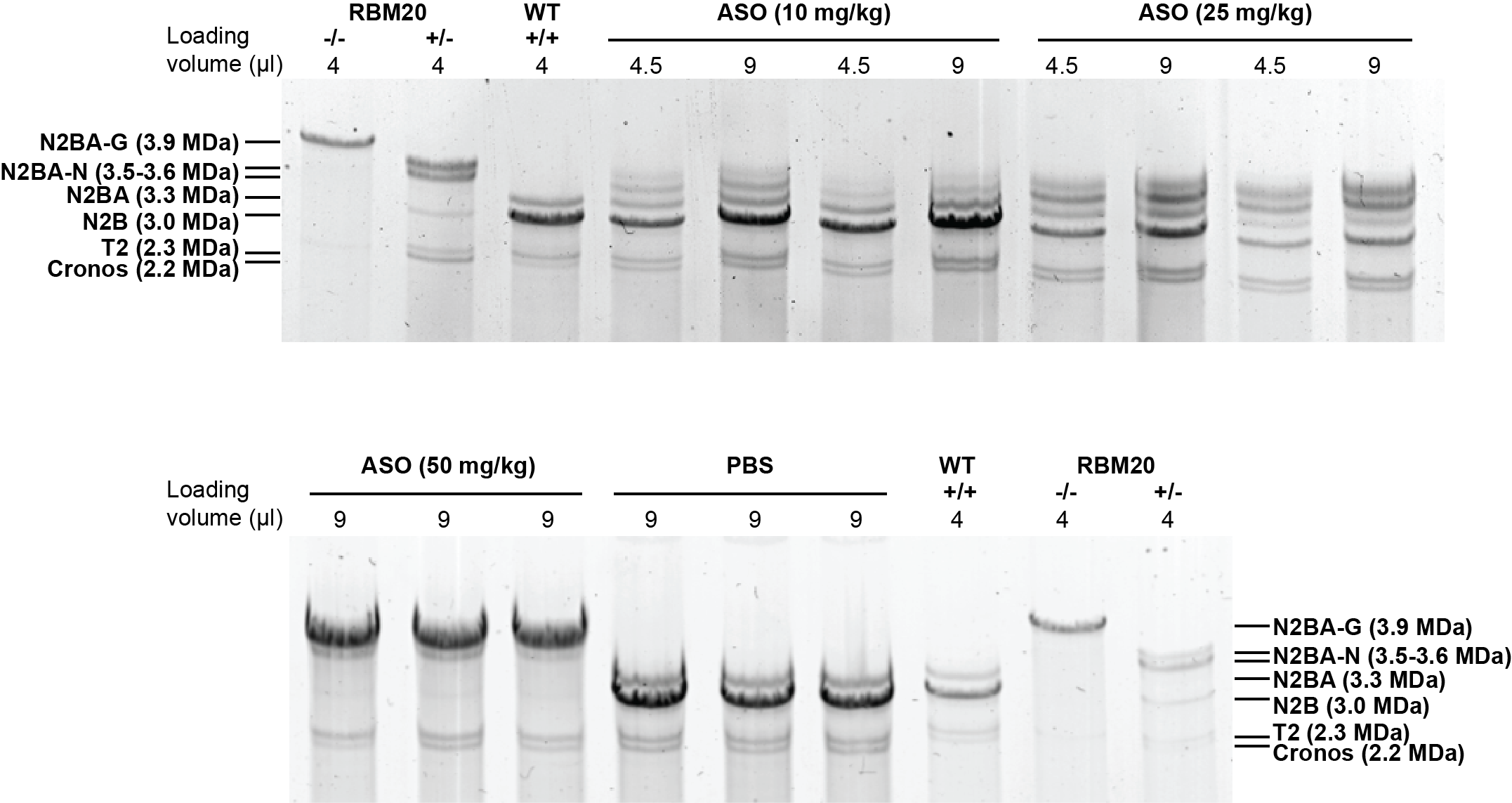
*

**Supplementary Figure 1.** Titin isoform expression in mouse LV myocardium following eight weekly injections of Rbm20-ASOs at doses of 10 mg/kg, 25 mg/kg, and 50 mg/kg. ASO at 10 mg/kg led to a slower upregulation of N2BA-N titin that did not reach 50% by the end of week 8 (upper panel), while ASO at 50 mg/kg resulted in an isoform switching to N2BA-G at the end of week 8 (lower panel). N2BA-G is the largest full-length isoform incorporating both the cardiac‐specific N2B unique element and extended PEVK regions. N2BA-N is a slightly smaller variant lacking portions of the PEVK region. N2BA is the wildtype isoform containing both N2B and N2A regions. N2B is the smaller full-length form containing only the shorter N2B‐unique element and minimal PEVK. Cronos is a short isoform arising from the internal Cronos promoter that lacks A- and M-band domains as well as main spring elements. T2 are the proteolytic C-terminal degradation products of full-length titin.

**
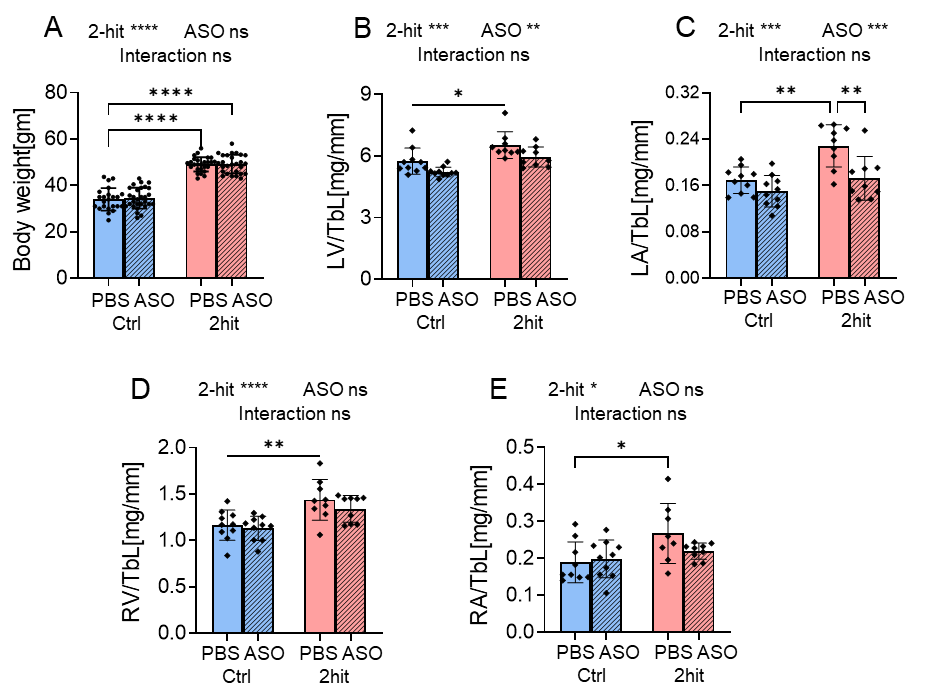
**

**Supplementary Figure 2.** Body weight and cardiac morphometry of 2-hit mice after ASO treatment. A) Body weight. B) The weights of the left ventricle (LV), C) left atrium (LA), D) right ventricle (RV), E) right atrium (RA) were normalized to tibial lengths. The data is presented as means ± SD. ns indicates non statistically significant. For simplicity, the comparison between both groups of the 2-hit vs the Ctrl-ASO are not displayed. Significance levels are indicated as * p≤0.05; **p≤0.01, ***p≤0.001; ****p≤0.0001. The sample sizes included n=10,10,9,9 mice for Ctrl-PBS, Ctrl-ASO, 2-hit-PBS, and 2-hit-ASO, respectively. Analysis was conducted using Two-way ANOVA with Tukey’s multiple comparisons. Two-way ANOVA analysis results are shown above each figure. Additional detailed parameters are available in Supplementary Table 3.


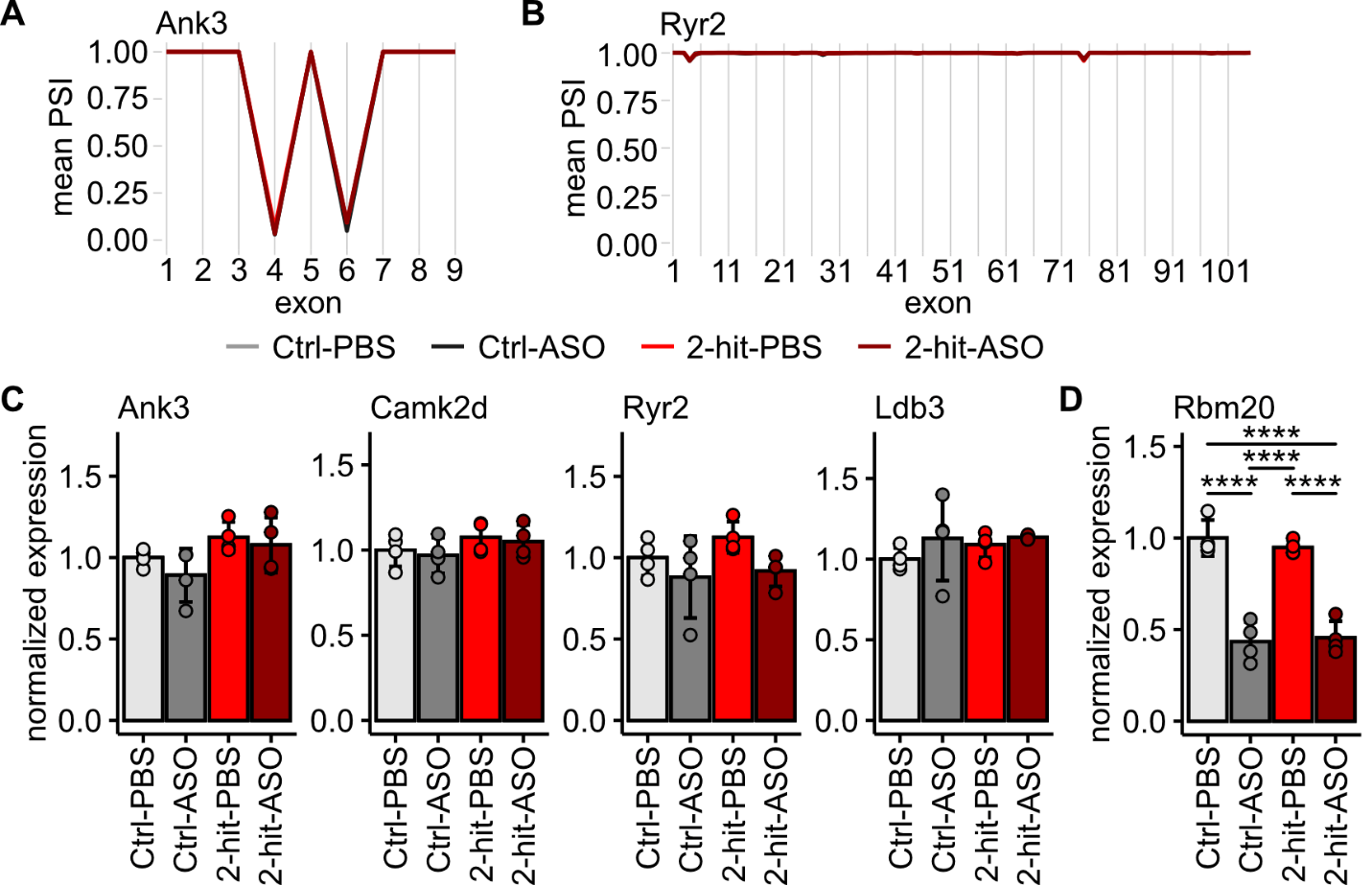


**Supplementary Figure 3.** The splicing of Ank3 and Ryr2, and the gene expression levels of Ank3, Camk2d, Ryr2, and Ldb3 remain unchanged after ASO treatment. A) PSI blot of Ank3 and B) Ryr2. C) Expression level of Ank3, CamK2d, Ryr2 and Ldb3. Not significant in two way ANOVA. D) Normalized Rbm20 gene expression levels. Significance levels are indicated as ****p≤0.0001. Analysis was conducted using Two-way ANOVA with Tukey’s multiple comparisons.

**
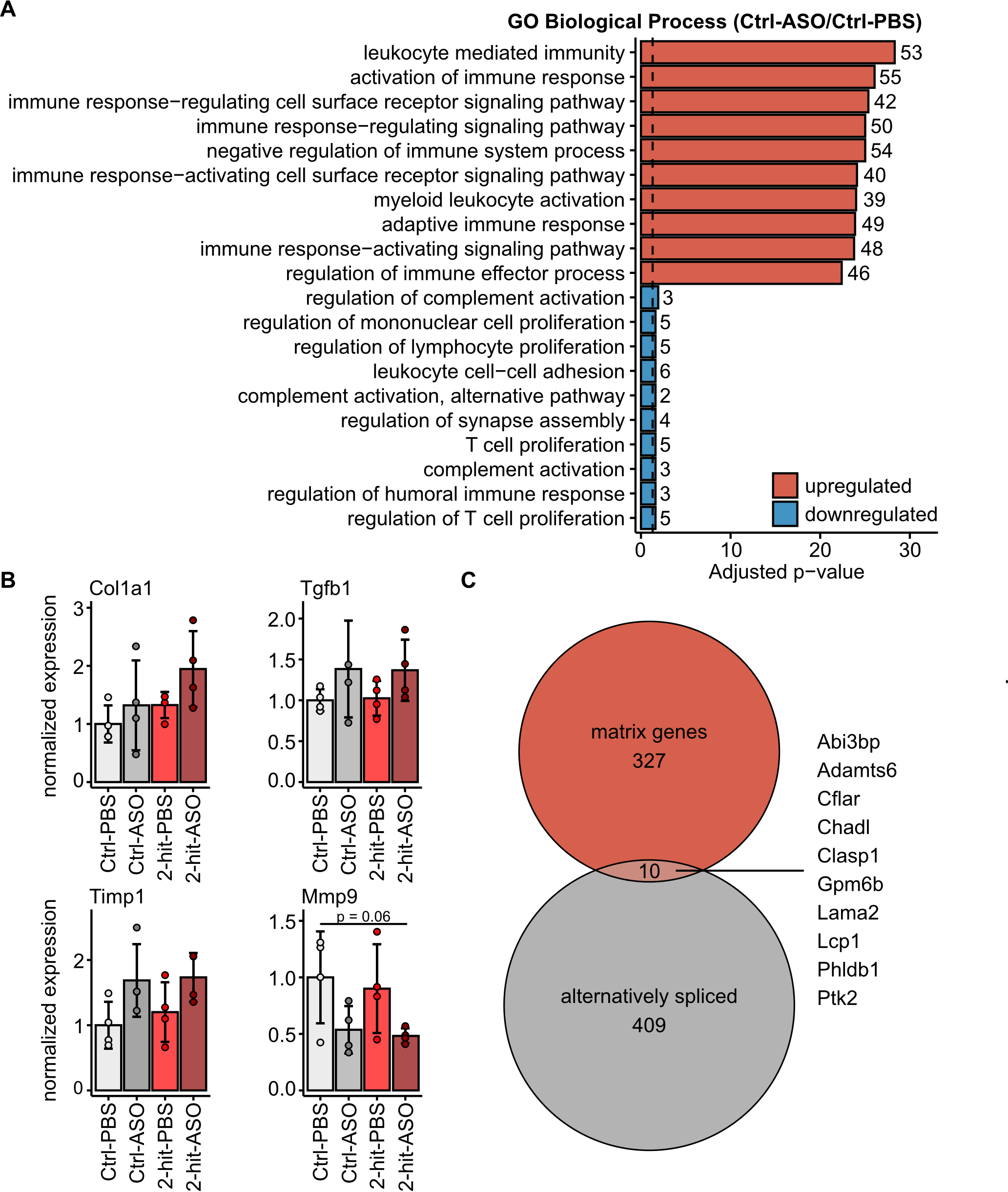
**

**Supplementary Figure 4.** A) Rbm20-ASOs induce differential expression of genes associated with immune response. B) Expression levels of fibrosis-related genes Col1a1, Tgfb1, Timp1, Mmp9. C) 10/337 matrix-related genes (GO:0030198) are differentially spliced (skipped exon event). Statistical analysis was carried out using Two-way-ANOVA with Tukey’s multiple comparisons.


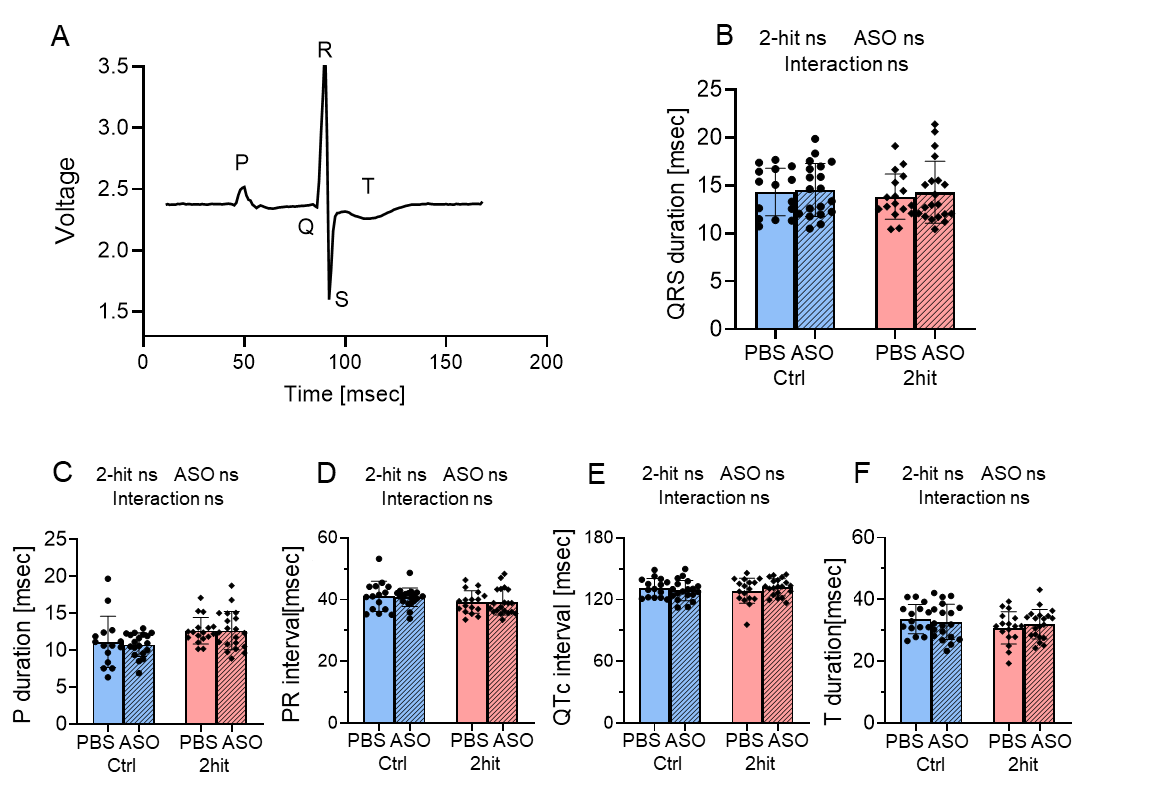


**Supplementary Figure 5.** Rbm20-ASOs do not induce any changes in electrocardiographic (ECG) parameters. A) ECG recordings were conducted under isoflurane anesthesia, with an example showing P, Q, R, S, and T waves. B) No significant differences are observed across the groups in QRS duration, C) P wave duration, D) PR interval, E) QTc duration, or F) T wave duration. The data is presented as means ± SD. ns indicates non statistically significant. Statistical analysis was carried out using Two-way ANOVA with Tukey's multiple comparisons. Two-way ANOVA analysis results are shown above each figure. Each data point represents an average from a single animal, with around 400 heartbeats analyzed per mouse. The study included n=15,19,17,20 mice for Ctrl-PBS, Ctrl-ASO, 2-hit-PBS, and 2-hit-ASO, respectively.

**Supplementary Tables**

**Supplementary Table1.** Echocardiographic parameters were compared in 2-hit mice at two time points: before the start of ASO treatment (16 weeks in 2-hit regimen) and after a 6-week ASO treatment period (22 weeks in 2-hit regimen).

| **Echocardiography** | Ctrl-PBS | | Ctrl-ASO | | 2-hit-PBS | | 2-hit-ASO | | Statistics (3way-ANOVA with RM or Mixed-effects model, matching by time) | | | | | | |
| --- | --- | --- | --- | --- | --- | --- | --- | --- | --- | --- | --- | --- | --- | --- | --- |
| Number of mice | 23 | | 28 | | 24 | | 25 | |  |  |  |  |  |  |  |
| timepoint | before Rx | after Rx | before Rx | after Rx | before Rx | after Rx | before Rx | after Rx | Time | 2-hit | ASO | Time  x  2-hit | Time  x  ASO | 2-hit  x  ASO | Time  x  2-hit  x  ASO |
| BW (gm) | 32.7±3.9 | 34±4.8 | 33.9±3.6 | 34.5±4.5 | 47.2±2.1 | 49.1±3.1 | 46.9±3.1 | 49.2±4.0 | **** | **** | ns | ns | ns | ns | ns |
| Heart Rate (bpm) | 495±52 | 509±47 | 482±61 | 504±48 | 510±46 | 500±35 | 474±50 | 505±47 | ** | ns | ns | ns | * | ns | ns |
| EF (%) | 44±7.6 | 45.1±6.9 | 41.4±5.2 | 40.2±8.3 | 48.6±8.4 | 48.5±6.5 | 47.7±5.8 | 44.1±7.0 | ns | *** | ** | ns | 0.056 | ns | ns |
| LV EDV (μL) | 88.2±13 | 88.9±14 | 92.2±14 | 87.5±14.8 | 84.2±11.2 | 86.4±11.9 | 82.3±12.1 | 89.8±16 | ns | ns | ns | ** | ns | ns | * |
| Stroke volume (μL) | 38.5±7.0 | 39.6±6.6 | 37.9±6.3 | 34.6±7.0 | 40.9±8.7 | 41.9±8.2 | 39±5.1 | 39.1±7.0 | ns | * | * | ns | 0.086 | ns | ns |
| WT;d (mm) | 0.63±0.07 | 0.66±0.06 | 0.63±0.06 | 0.62±0.05 | 0.74±0.08 | 0.76±0.07 | 0.71±0.06 | 0.74±0.08 | * | **** | ns | ns | ns | ns | ns |
| WT;s (mm) | 0.92±0.12 | 0.95±0.10 | 0.88±0.08 | 0.87±0.10 | 1.04±0.14 | 1.06±0.10 | 1.0±0.07 | 1.02±0.11 | ns | **** | ** | ns | ns | ns | ns |
| LA dimension [mm] | 2.23±0.18 | 2.25±0.25 | 2.17±0.17 | 2.2±0.18 | 2.54±0.29 | 2.62±0.28 | 2.47±0.19 | 2.48±0.23 | ns | **** | * | ns | ns | ns | ns |
| LV MPI | 0.64±0.10 | 0.63±0.12 | 0.66±0.12 | 0.68±0.12 | 0.68±0.12 | 0.71±0.10 | 0.71±0.15 | 0.73±0.15 | ns | ** | ns | ns | ns | ns | ns |
| Eccentricity | 3.53±0.42 | 3.4±0.43 | 3.61±0.46 | 3.56±0.46 | 2.96±0.40 | 2.92±0.33 | 3.03±0.34 | 3.03±0.44 | ns | **** | ns | ns | ns | ns | ns |
| E decel time (ms) | 23.3±3.6 | 24.2±3.2 | 23.9±3.2 | 23.8±3.2 | 20.4±3.5 | 19.8±2.6 | 20.5±3.7 | 22.7±4.3 | ns | **** | ns | ns | ns | ns | * |
| MV e' (mm/sec) | -18.7±3.6 | -17.8±2.9 | -16.7±3.1 | -17.7±3.6 | -17.6±4.3 | -15.4±4.1 | -15.8±3.2 | -16.9±3.6 | ns | * | ns | ns | ** | ns | ns |
| MV E/A | 1.7±0.6 | 1.6±0.3 | 1.7±0.4 | 1.4±0.3 | 1.8±0.4 | 2±0.6 | 1.9±0.5 | 1.6±0.5 | ns | ** | ns | ns | * | ns | ns |
| MV E/e´ | -30.9±4.4 | -32.2±6.1 | -33.9±5.5 | -31.1±6.4 | -33.9±7.8 | -38.5±11 | -35.9±7.0 | -30.2±6.3 | ns | * | ns | ns | *** | 0.057 | ns |

BW (body weight), EF (ejection fraction), LV (left ventricle), LVEDV (LV end-diastolic volume), WT;d (wall thickness in diastole), WT;s (wall thickness in systole), LA (left atrium), MPI (myocardial performance index), Eccentricity (LVID;d / (LVPW;d + LVAW;d)), E decel time (Mitral E deceleration time), MV (mitral valve), E (early diastolic filling), A (late diastolic filling), and e’ (early diastolic velocity of mitral annulus movement). The data is presented as means ± SD. Statistical significance is indicated as * for p≤0.05, ** for p≤0.01, *** for p≤0.001, **** for p≤0.0001. The analysis was conducted using either Three-way ANOVA with repeated measurement or a Mixed-effects model, matched by time factor, with post hoc testing using Tukey's method.

**Supplementary Table 2.** Pressure volume analysis parameters were assessed in 2-hit mice following ASO/PBS treatment.

| **Pressure-Volume analysis** | Ctrl-PBS | Ctrl-ASO | 2-hit-PBS | 2-hit-ASO | Two-way ANOVA | | |
| --- | --- | --- | --- | --- | --- | --- | --- |
|  |  |  |  |  | Inter-action | ASO | 2-hit |
| Animals measured | n=11 | n=11 | n=11 | n=10 |  |  |  |
| *Load Dependent Parameters* |  |  |  |  |  |  |  |
| ESP (mmHg) | 83.8±12.7 | 70.4±6.7 *^, ƗƗƗƗ^ | 102.9±10.9 ** | 93.7±11.6 | ns | ** | **** |
| EDP (mmHg) | 3.9±1.9 | 2.6±1.6 ^ƗƗ^ | 5.9±2.9 | 3.1±2.5 ^Ɨ^ | ns | ** | ns |
| dPmax (mmHg/s) | 6400±1973 | 5435±1109 | 7457±2001 | 7168±1478 | ns | ns | * |
| dPmin (mmHg/s) | -6018±1973 | -4876±914 | -6513±1883 | -6667±1415 | ns | ns | * |
| SV (µl) | 39.3±3.9 | 41.3±4.8 | 39±4.2 | 43±8.3 | ns | ns | ns |
| CO (mL/min) | 16.4±2.5 | 17.6±3.0 | 16.9±2.7 | 17.6±4.0 | ns | ns | ns |
| Tau Logistic (ms) | 15.2±3.4 | 16.0±3.0 | 19.4±4.2 * | 15.8±3.0 | * | ns | ns |
| Tau glantz (ms) | 28.7±5.4 | 27.6±5.8 | 32.5±6.8 | 26.5±5.4 | ns | ns | ns |
| Tau Weiss (ms) | 8.1±2.2 | 8.4±1.5 | 8.9±2.6 | 7.8±1.5 | ns | ns | ns |
| Ea (mmHg/µl) | 2.2±0.4 | 1.7±0.3 ^ƗƗƗƗ^ | 2.7±0.5 * | 2.3±0.4 | ns | *** | *** |
| Ventricular-arterial coupling | 0.82±0.3 | 0.72±0.2 | 0.63±0.24 | 0.66±0.21 | ns | ns | ns |
| *Load Independent Parameters* |  |  |  |  |  |  |  |
| ESPVR(mmHg/µl) | 2.963±0.90 | 2.568±0.762 ^ƗƗ^ | 4.69±1.541** | 3.412±1.45 | ns | * | ** |
| PRSW (mmHg) | 72.1±21.5 | 61.96±8.66^ƗƗƗ^ | 93.05±20.78* | 70.46±8.96 ^Ɨ^ | ns | ** | ** |
| EDPVR (mmHg/µl) | 0.024±0.014 | 0.017±0.007 ^ƗƗƗ^ | 0.056±0.025**** | 0.023±0.015 ^ƗƗƗ^ | * | *** | *** |

ESP (end-systolic pressure), EDP (end-diastolic pressure), dPmax (the maximum rate of pressure rise), dPmin (the maximum rate of pressure decrease), SV (stroke volume), CO (cardiac output), Tau (LV relaxation–time constant), Ea (effective arterial elastance), ESPVR (end-systolic pressure-volume relation), PRSW (preload recruitable stroke work), EDPVR (end-diastolic pressure-volume relation).The data is presented as means ± SD. Statistical significance is denoted by * p≤0.05, ** p≤0.01, *** p≤0.001, **** p≤0.0001 as significant versus Ctrl-PBS, and Ɨ p≤0.05,ƗƗ p≤0.01, ƗƗƗ p≤0.001, ƗƗƗƗ p≤0.0001 as significant versus 2-hit-PBS. The analysis was conducted using Two-way ANOVA with Tukey’s multiple comparisons.

**Supplementary Table 3.** Tissue Morphometry were assessed in 2-hit mice following ASO/PBS treatment.

| **Tissue Morphometry** | Ctrl-PBS | Ctrl-ASO | 2-hit-PBS | 2-hit-ASO | Two-way ANOVA | | |
| --- | --- | --- | --- | --- | --- | --- | --- |
|  |  |  |  |  | Inter-action | ASO | 2-hit |
| Animals measured | n=10 | n=10 | n=9 | n=9 |  |  |  |
| LV/TbL (mg/mm) | 5.76±0.64 | 5.23±0.23 ^ƗƗƗƗ^ | 6.53±0.65* | 5.96±0.49 | ns | ** | *** |
| RV/TbL (mg/mm) | 1.17±0.16 | 1.13±0.13 ^ƗƗ^ | 1.44±0.22** | 1.34±0.14 | ns | ns | **** |
| LA/TbL(mg/mm) | 0.17±0.02 | 0.15±0.03 ^ƗƗƗƗ^ | 0.23±0.04** | 0.17±0.04 ^ƗƗ^ | ns | *** | *** |
| RA/TbL(mg/mm) | 0.19±0.06 | 0.2±0.05 | 0.27±0.08* | 0.22±0.02 | ns | ns | * |
| Lung water  (wet/dry wt) | 4.56±0.20 | 4.63±0.30 | 4.37±0.22 | 4.41±0.22 | ns | ns | ** |
| spleen weight/TbL (mg/mm) | 4.3±0.6 | 7.6±3.7 | 5.2±1.2 | 10.3±6.8***^,ƗƗƗ^ | ns | **** | * |
| kidney weight/TbL (mg/mm) | 20.7±2.4 | 20.9±1.8 ^Ɨ^ | 23.0±2.3** | 22.0±1.5 | ns | ns | ** |
| Liver weight/TbL  (mg/mm) | 76.5±20.4 | 99.0±16.2* | 107.2±21.4**** | 121.3±21.8**** | ns | *** | **** |

LV (left ventricle), TL (tibial length), RV (right ventricle), LA (left atrium), and RA (right atrium). The data is presented as means ± SD. Statistical significance is denoted by * p≤0.05, **,p≤0.01, ***p≤0.001, ****p≤0.0001 as significant versus Ctrl-PBS, and Ɨ p≤0.05, ƗƗ p≤0.01, ƗƗƗp≤0.001, ƗƗƗƗ p≤0.0001 as significant versus 2-hit-PBS. The analysis was conducted using Two-way ANOVA with Tukey’s multiple comparisons.

**Supplementary Table 4.** Intact cardiomyocyte parameters.

| **Intact cardiomyocyte** | Ctrl-PBS | Ctrl-ASO | 2-hit-PBS | 2-hit-ASO | Two-way ANOVA | | |
| --- | --- | --- | --- | --- | --- | --- | --- |
|  |  |  |  |  | Inter-action | ASO | 2-hit |
| number of mice | n=7 | n=7 | n=8 | n=9 |  |  |  |
| **Unloaded parameters** |  |  |  |  |  |  |  |
| Baseline SL [µm] | 1.76±0.01 | 1.77±0.02 | 1.74±0.02 | 1.75±0.04 | ns | ns | * |
| Transient amplitude[mN/mm^2^] | 0.19±0.02 | 0.18±0.02 | 0.18±0.03 | 0.18±0.02 | ns | ns | ns |
| Time to 50% peak stress [msec] | 16.3±1.44 | 17.18±0.59 | 17.07±1.07 | 16.83±0.92 | ns | ns | ns |
| Time to peak transient[msec] | 46.53±4.53 | 44.55±2.45 | 44.7±1.98 | 43.3±0.93 | ns | ns | ns |
| Time to 50% stress decay[msec] | 18.68±1.53 | 17.48±1.23 | 18.15±1.71 | 17.97±1.27 | ns | ns | ns |
| Time to 90% stress decay[msec] | 37.26±1.98 | 38.25±1.85 | 36.41±2.16 | 37.88±1.51 | ns | ns | ns |
| **Loaded parameters** |  |  |  |  |  |  |  |
| Stress amplitude [mN/mm^2^] | 1.33±0.27 | 1.39±0.24 | 1.65±0.26 | 1.39±0.25 | ns | ns | ns |
| ED-SSLR  [mN/mm^2^.μm] | 8.45±0.76 | 8.81±1.80 ^ƗƗƗƗ^ | 16.54±3.89**** | 10.88±2.39 ^ƗƗƗ^ | ** | ** | **** |
| ED-SSLR X intercept [μm] | 1.81±0.05 | 1.85±0.04 | 1.84±0.03 | 1.85±0.04 | ns | ns | ns |
| ES-SSLR  [mN/mm^2^.μm] | 26.16±3.94 | 22.73±3.32 ^ƗƗƗƗ^ | 43.19±10.37*** | 28.65±6.18 ^ƗƗƗ^ | * | *** | **** |
| ES-SSLR X intercept[μm] | 1.74±0.06 | 1.75±0.06 | 1.78±0.05 | 1.76±0.04 | ns | ns | ns |
| PRSW[mN/mm^2^] | 2.15±0.59 | 2.08±0.50 | 2.26±0.34 | 1.9±0.42 | ns | ns | ns |
| Stroke work [mN] | 9.58±3.43 | 10.63±3.66 | 9.22±4.04 | 11.7±4.61 | ns | ns | ns |
| Stroke length [ΔSL] | 0.033±0.007 | 0.04±0.01 | 0.027±0.01 | 0.037±0.011 | ns | * | ns |
| Tau Logistic [msec] | 16.89±3.40 | 17.06±4.27 | 19.74±5.53 | 17.18±3.70 | ns | ns | ns |

SL (sarcomere length), ED-SSLR (end diastolic stress-sarcomere length relation), ES-SSLR (end systolic stress-sarcomere length relation), PRSW (preload recruitable stroke work). The data is presented as means ± SD. Statistical significance is denoted by * p≤0.05, ** p≤0.01, *** p≤0.001, **** p≤0.0001 as significant versus Ctrl-PBS, and Ɨ p≤0.05, ƗƗ p≤0.01, ƗƗƗ p≤0.001, ƗƗƗƗ p≤0.0001 as significant versus 2-hit-PBS. The analysis was conducted using Two-way ANOVA with Tukey’s multiple comparisons. Data of 4-8 cells were analyzed per mouse.

**References**

1. Pepin ME, Konrad P, Nazir S, Dewenter M, Backs J. Disrupting Nicotinamide Nucleotide Transhydrogenase Prevents Cardiometabolic Heart Failure With Preserved Ejection In Mice. *Circulation Research* 2023;**133**.

2. Schiattarella GG, Altamirano F, Tong D, French KM, Villalobos E, Kim SY, Luo X, Jiang N, May HI, Wang ZV, Hill TM, Mammen PPA, Huang J, Lee DI, Hahn VS, Sharma K, Kass DA, Lavandero S, Gillette TG, Hill JA. Nitrosative stress drives heart failure with preserved ejection fraction. *Nature* 2019;**568**:351-356.

3. Radke MH, Badillo-Lisakowski V, Britto-Borges T, Kubli DA, Juttner R, Parakkat P, Carballo JL, Huttemeister J, Liss M, Hansen A, Dieterich C, Mullick AE, Gotthardt M. Therapeutic inhibition of RBM20 improves diastolic function in a murine heart failure model and human engineered heart tissue. *Sci Transl Med* 2021;**13**:eabe8952.

4. Warren CM, Jordan MC, Roos KP, Krzesinski PR, Greaser ML. Titin isoform expression in normal and hypertensive myocardium. *Cardiovascular research* 2003;**59**:86-94.

5. Warren CM, Krzesinski PR, Greaser ML. Vertical agarose gel electrophoresis and electroblotting of high-molecular-weight proteins. *Electrophoresis* 2003;**24**:1695-1702.

6. Hudson BD, Hidalgo CG, Gotthardt M, Granzier HL. Excision of titin's cardiac PEVK spring element abolishes PKCalpha-induced increases in myocardial stiffness. *J Mol Cell Cardiol* 2010;**48**:972-978.

7. Burkhoff D, Mirsky I, Suga H. Assessment of systolic and diastolic ventricular properties via pressure-volume analysis: a guide for clinical, translational, and basic researchers. *Am J Physiol Heart Circ Physiol* 2005;**289**:H501-512.

8. O'Connell TD, Rodrigo MC, Simpson PC. Isolation and culture of adult mouse cardiac myocytes. *Methods Mol Biol* 2007;**357**:271-296.

9. Helmes M, Najafi A, Palmer BM, Breel E, Rijnveld N, Iannuzzi D, van der Velden J. Mimicking the cardiac cycle in intact cardiomyocytes using diastolic and systolic force clamps; measuring power output. *Cardiovasc Res* 2016;**111**:66-73.

10. Methawasin M, Strom J, Borkowski T, Hourani Z, Runyan R, Smith JE, 3rd, Granzier H. Phosphodiesterase 9a Inhibition in Mouse Models of Diastolic Dysfunction. *Circ Heart Fail* 2020;**13**:e006609.

11. Granzier HL, Irving TC. Passive tension in cardiac muscle: contribution of collagen, titin, microtubules, and intermediate filaments. *Biophys J* 1995;**68**:1027-1044.

12. Aschar-Sobbi R, Izaddoustdar F, Korogyi AS, Wang Q, Farman GP, Yang F, Yang W, Dorian D, Simpson JA, Tuomi JM, Jones DL, Nanthakumar K, Cox B, Wehrens XH, Dorian P, Backx PH. Increased atrial arrhythmia susceptibility induced by intense endurance exercise in mice requires TNFalpha. *Nat Commun* 2015;**6**:6018.

13. Irving TC, Konhilas J, Perry D, Fischetti R, de Tombe PP. Myofilament lattice spacing as a function of sarcomere length in isolated rat myocardium. *American journal of physiology Heart and circulatory physiology* 2000;**279**:H2568-2573.

14. Chen S, Zhou Y, Chen Y, Gu J. fastp: an ultra-fast all-in-one FASTQ preprocessor. *Bioinformatics* 2018;**34**:i884-i890.

15. Dobin A, Davis CA, Schlesinger F, Drenkow J, Zaleski C, Jha S, Batut P, Chaisson M, Gingeras TR. STAR: ultrafast universal RNA-seq aligner. *Bioinformatics* 2013;**29**:15-21.

16. Li H, Handsaker B, Wysoker A, Fennell T, Ruan J, Homer N, Marth G, Abecasis G, Durbin R, Genome Project Data Processing S. The Sequence Alignment/Map format and SAMtools. *Bioinformatics* 2009;**25**:2078-2079.

17. Love MI, Huber W, Anders S. Moderated estimation of fold change and dispersion for RNA-seq data with DESeq2. *Genome Biol* 2014;**15**:550.

18. Shen S, Park JW, Lu ZX, Lin L, Henry MD, Wu YN, Zhou Q, Xing Y. rMATS: robust and flexible detection of differential alternative splicing from replicate RNA-Seq data. *Proc Natl Acad Sci U S A* 2014;**111**:E5593-5601.

19. Yu G, Wang LG, Han Y, He QY. clusterProfiler: an R package for comparing biological themes among gene clusters. *OMICS* 2012;**16**:284-287.

20. Schafer S, Miao K, Benson CC, Heinig M, Cook SA, Hubner N. Alternative Splicing Signatures in RNA-seq Data: Percent Spliced in (PSI). *Curr Protoc Hum Genet* 2015;**87**:11 16 11-11 16 14.
