# Supplementary material for: Rbm20 antisense oligonucleotides alleviate diastolic dysfunction in a mouse model of cardiometabolic heart failure (HFpEF)
Source: Cardiovasc Res. 2025 Oct 17;121(13):2027–41. doi: 10.1093/cvr/cvaf171 (PMC12560776; doi:10.1093/cvr/cvaf171)
Supplement: cvaf171_Supplementary_Data [file cvaf171_supplementary_data.zip › Uncropped Gels+Blots.pdf]

Uncropped titin isoform analysis of Figure 1B, at 0 and 2 weeks

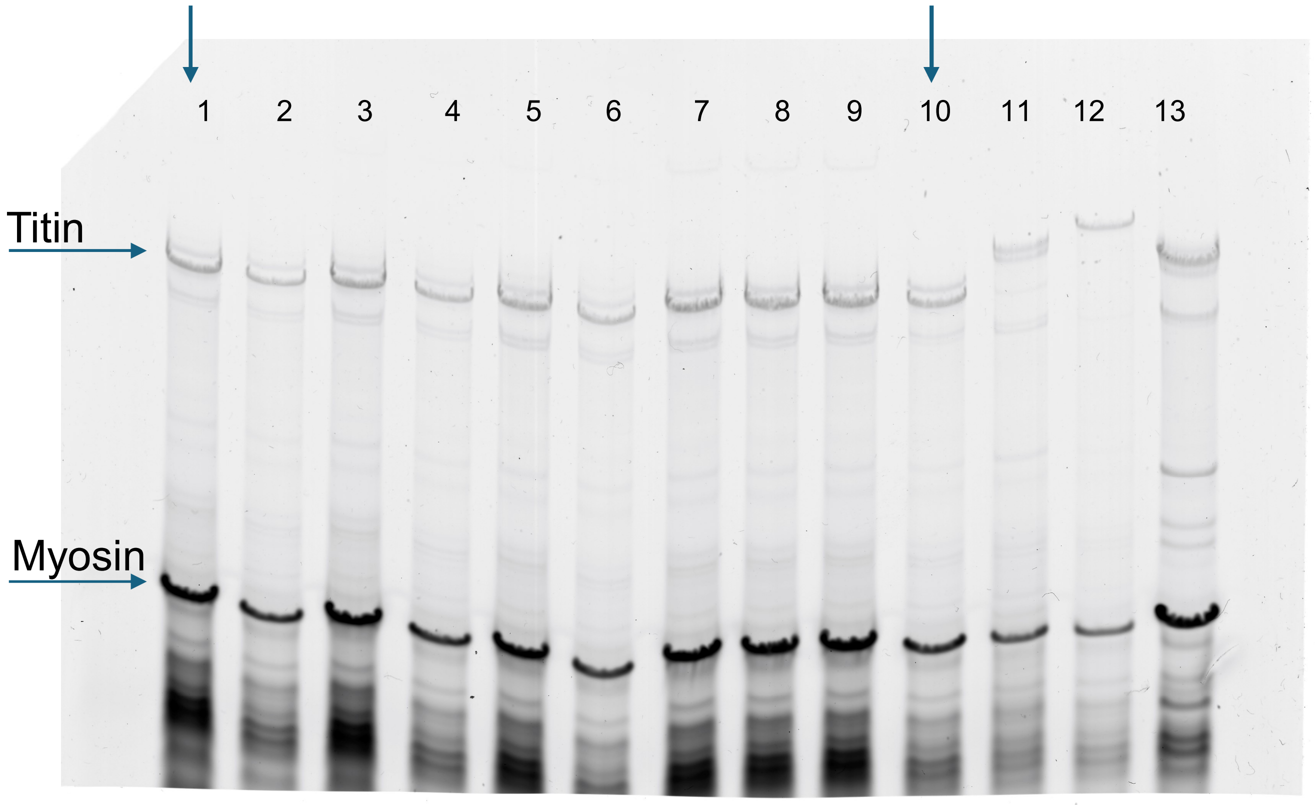

Lane	Sample ID	Muscle	Strain	Genotyp e	Treatment	Vol (uL)
1	100-2210 LP	LV	Black6N	WT	ASO 25mg/kg 2 doses	9
2	100-2210 LP	LV	Black6N	WT	ASO 25mg/kg 2 doses	4.5
3	100-2210 NP	LV	Black6N	WT	ASO 25mg/kg 2 doses	9
4	100-2211 NP	LV	Black6N	WT	ASO 25mg/kg 2 doses	4.5
5	100-2211 DLP	LV	Black6N	WT	ASO 25mg/kg 2 doses	9
6	100-2211 DLP	LV	Black6N	WT	ASO 25mg/kg 2 doses	4.5
7	100-2215 LP	LV	Black6N	WT	ASO 10mg/kg 2 doses	9
8	100-2215 RP	LV	Black6N	WT	ASO 10mg/kg 2 doses	9
9	100-2215 DLP	LV	Black6N	WT	ASO 10mg/kg 2 doses	9
10	M7363	LV	Black6	WT		4
11	d8 Het	LV	RBM20	Het		4
12	M7271	LV	RBM20	Hom		4
13	BL6 TC	TC	BL6	WT		4

Uncropped titin isoform analysis of Figure 1B, at 3 weeks

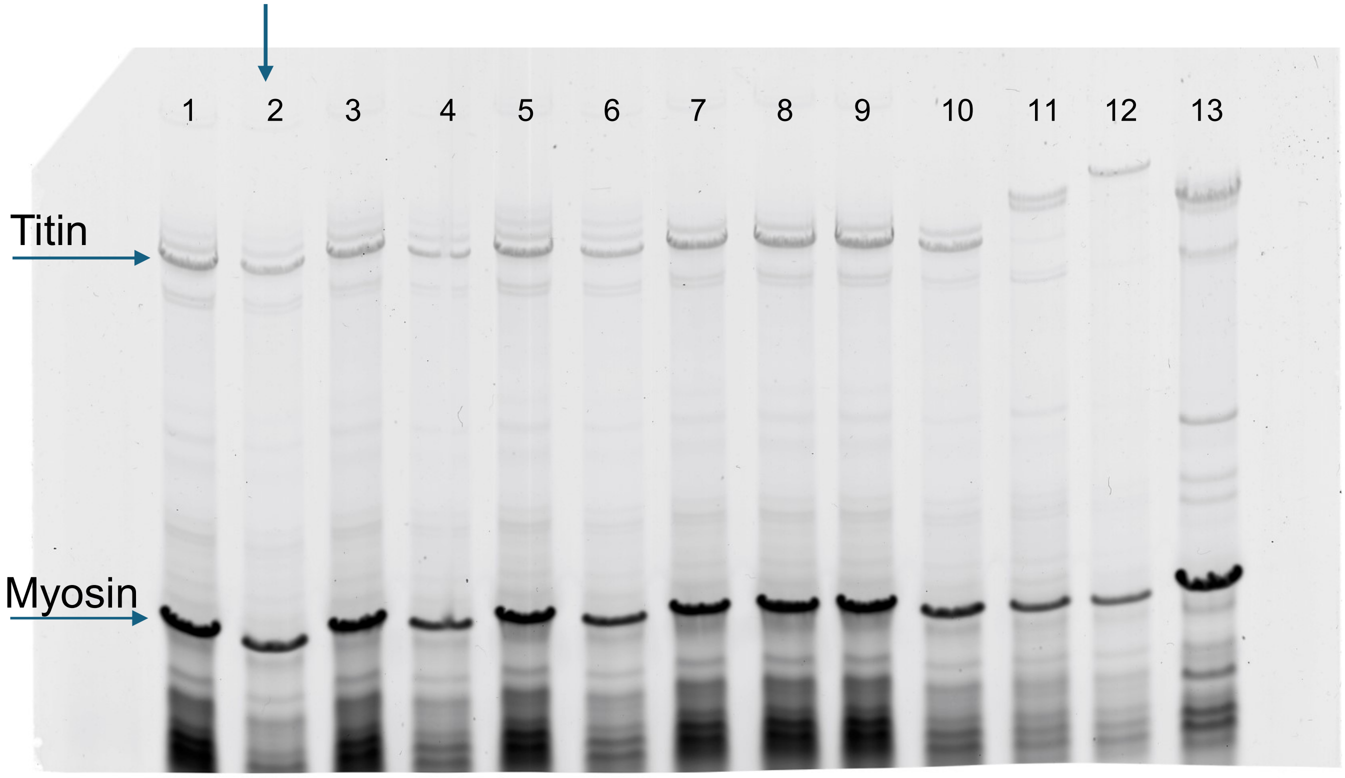

Lane	Sample ID	Muscle	Strain	Genotype	Treatment	Vol (uL)
1	100-2212 LP	LV	Black6N	WT	ASO 25mg/kg 3 doses	9
2	100-2212 LP	LV	Black6N	WT	ASO 25mg/kg 3 doses	4.5
3	100-2212 RP	LV	Black6N	WT	ASO 25mg/kg 3 doses	9
4	100-2212 RP	LV	Black6N	WT	ASO 25mg/kg 3 doses	4.5
5	100-2211 LP	LV	Black6N	WT	ASO 25mg/kg 3 doses	9
6	100-2211 LP	LV	Black6N	WT	ASO 25mg/kg 3 doses	4.5
7	100-2213 DRP	LV	Black6N	WT	ASO 10mg/kg 3 doses	9
8	100-2214 DLP	LV	Black6N	WT	ASO 10mg/kg 3 doses	9
9	100-2214 DRP	LV	Black6N	WT	ASO 10mg/kg 3 doses	9
10	M7363	LV	Black6	WT		4
11	d8 Het	LV	RBM20	Het		4
12	M7271	LV	RBM20	Hom		4
13	BL6 TC	TC	BL6	WT		4

Uncropped titin isoform analysis of Figure 1B, at 4 weeks

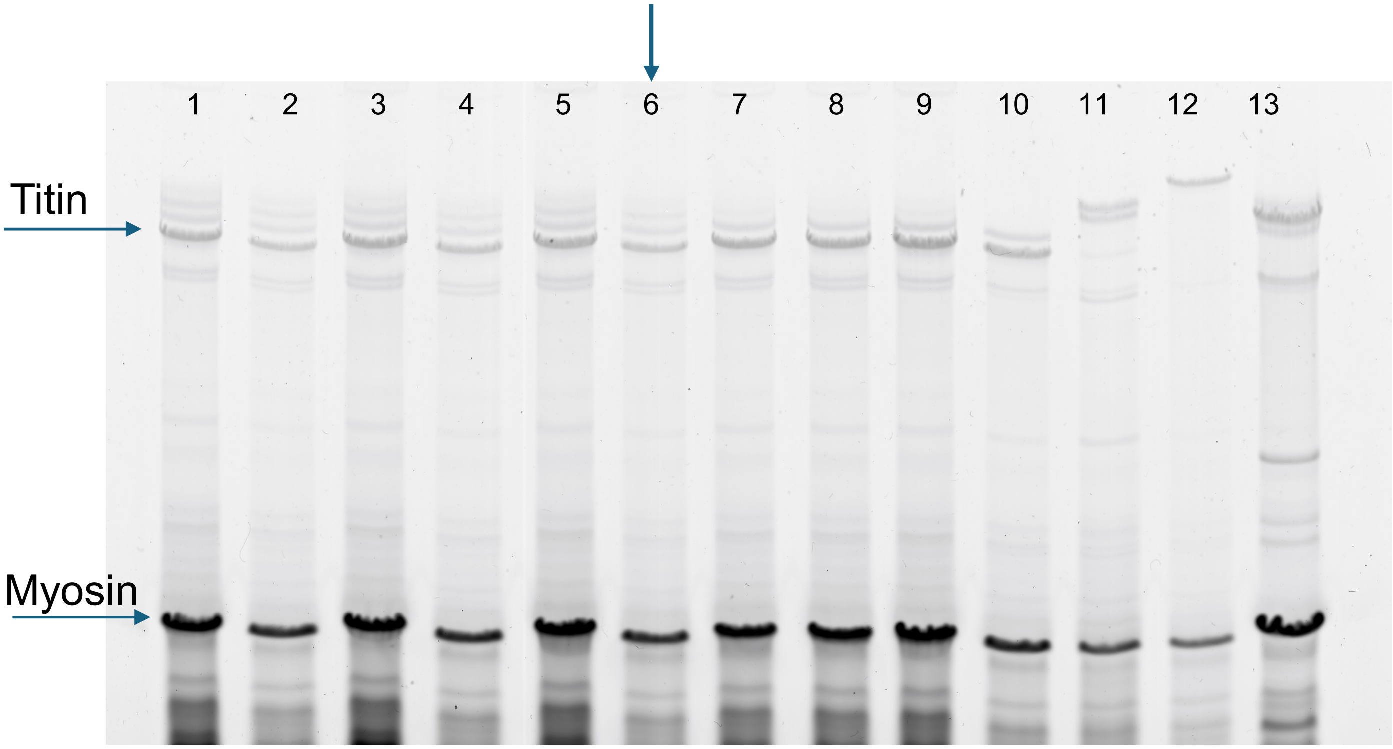

Lane	Sample ID	Muscle	Strain	Genotype	Treatment	Vol (uL)
1	100-2211 DRP	LV	Black6N	WT	ASO 25mg/kg 4 doses	9
2	100-2211 DRP	LV	Black6N	WT	ASO 25mg/kg 4 doses	4.5
3	100-2211 NP	LV	Black6N	WT	ASO 25mg/kg 4 doses	9
4	100-2211 NP	LV	Black6N	WT	ASO 25mg/kg 4 doses	4.5
5	100-2212 DLP	LV	Black6N	WT	ASO 25mg/kg 4 doses	9
6	100-2212 DLP	LV	Black6N	WT	ASO 25mg/kg 4 doses	4.5
7	100-2213 LP	LV	Black6N	WT	ASO 10mg/kg 4 doses	9
8	100-2214 RP	LV	Black6N	WT	ASO 10mg/kg 4 doses	9
9	100-2215 NP	LV	Black6N	WT	ASO 10mg/kg 4 doses	9
10	M7363	LV	Black6	WT		4
11	d8 Het	LV	RBM20	Het		4
12	M7271	LV	RBM20	Hom		4
13	BL6 TC	TC	BL6	WT		4

Uncropped titin isoform analysis of Figure 1B, at 5 weeks

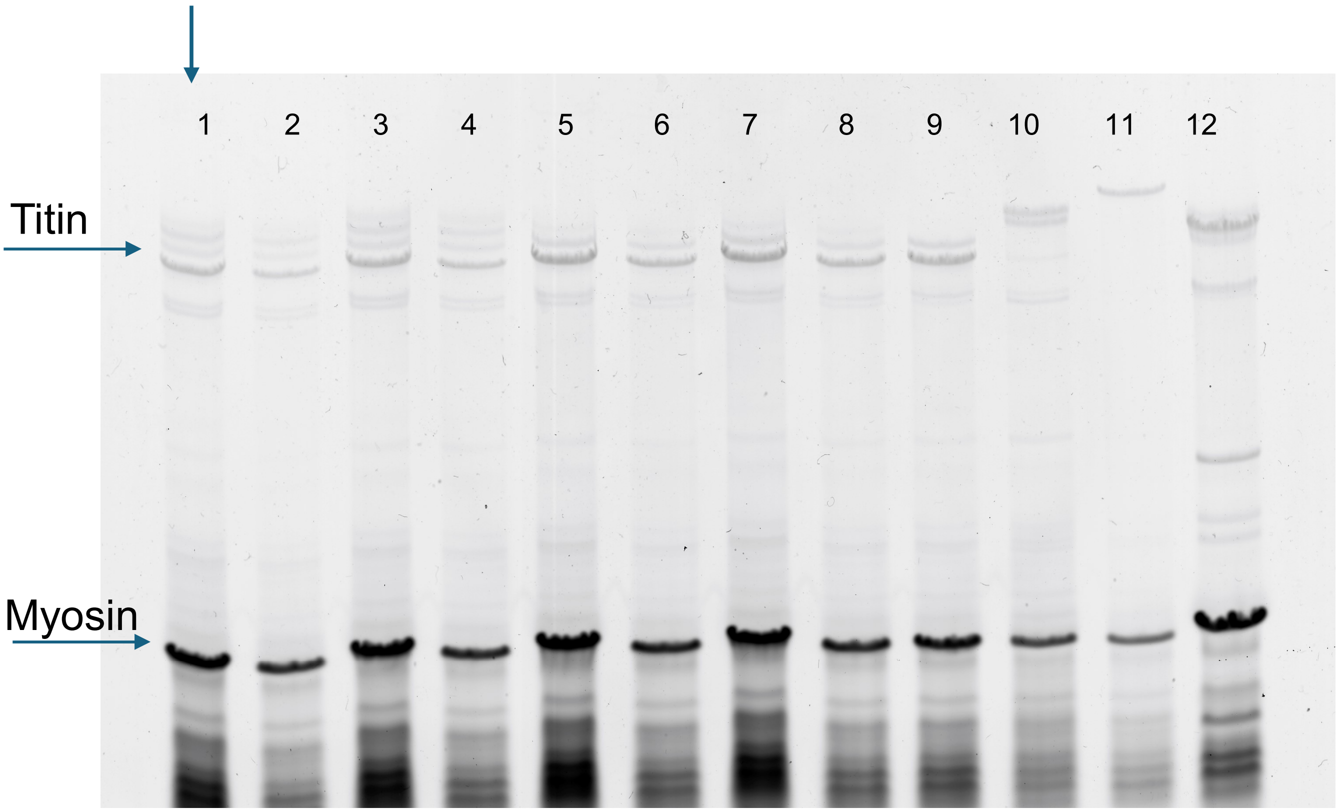

Lane	Sample ID	Muscle	Strain	Genotyp e	Treatment	Vol (uL)
1	100-2211 RP	LV	Black6N	WT	ASO 25mg/kg 5 doses	9
2	100-2211 RP	LV	Black6N	WT	ASO 25mg/kg 5 doses	4.5
3	100-2210 RP	LV	Black6N	WT	ASO 25mg/kg 5 doses	9
4	100-2210 RP	LV	Black6N	WT	ASO 25mg/kg 5 doses	4.5
5	100-2213 NP	LV	Black6N	WT	ASO 10mg/kg 5 doses	9
6	100-2213 NP	LV	Black6N	WT	ASO 10mg/kg 5 doses	4.5
7	100-2215 DRP	LV	Black6N	WT	ASO 10mg/kg 5 doses	9
8	100-2215 DRP	LV	Black6N	WT	ASO 10mg/kg 5 doses	4.5
9	M7363	LV	Black6	WT		4
10	d8 Het	LV	RBM20	Het		4
11	M7271	LV	RBM20	Hom		4
12	BL6 TC	TC	BL6	WT		4

Uncropped titin isoform analysis of Figure 1B, at 6 weeks

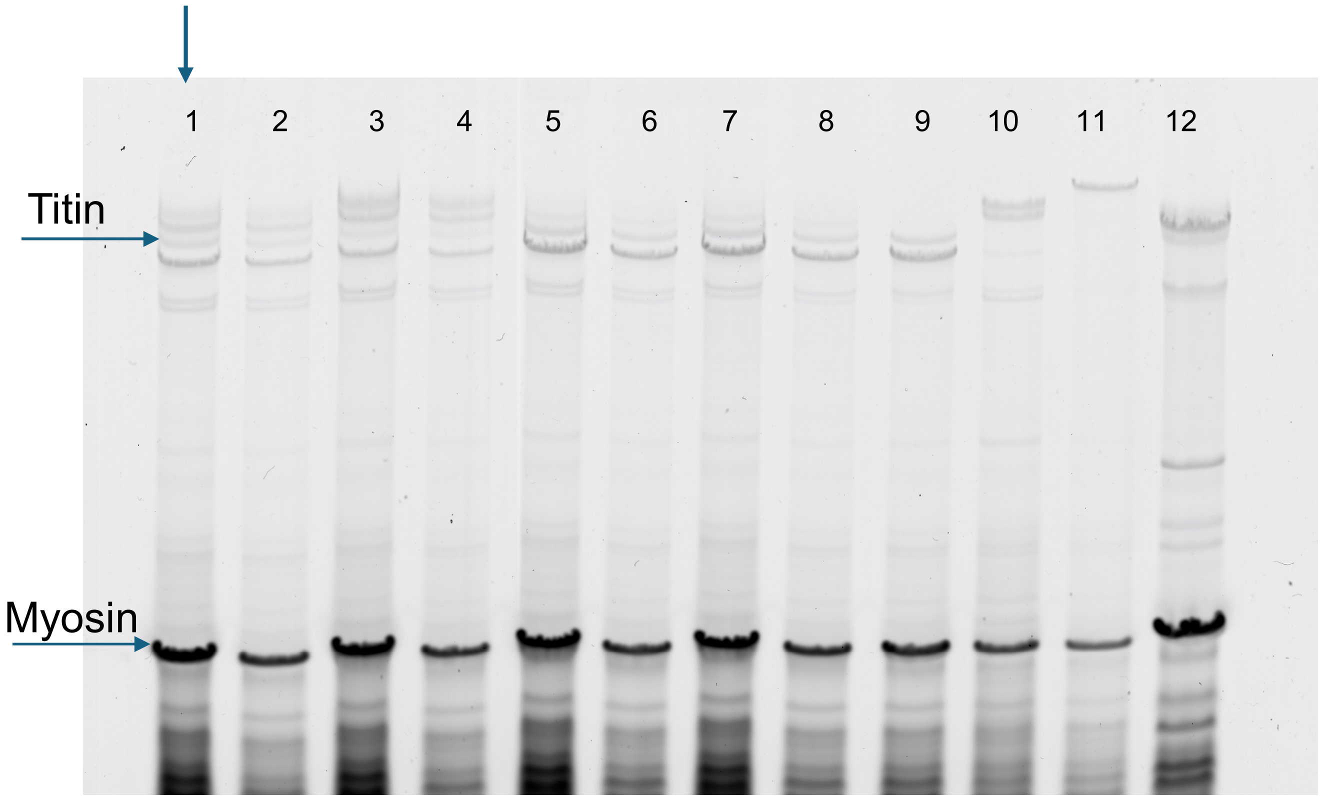

Lane	Sample ID	Muscle	Strain	Genotype	Treatment	Vol (uL)
1	100-2212 DRP	LV	Black6N	WT	ASO 25mg/kg 6 doses	9
2	100-2212 DRP	LV	Black6N	WT	ASO 25mg/kg 6 doses	4.5
3	100-2212 NP	LV	Black6N	WT	ASO 25mg/kg 6 doses	9
4	100-2212 NP	LV	Black6N	WT	ASO 25mg/kg 6 doses	4.5
5	100-2214 LP	LV	Black6N	WT	ASO 10mg/kg 6 doses	9
6	100-2214 LP	LV	Black6N	WT	ASO 10mg/kg 6 doses	4.5
7	100-2214 NP	LV	Black6N	WT	ASO 10mg/kg 6 doses	9
8	100-2214 NP	LV	Black6N	WT	ASO 10mg/kg 6 doses	4.5
9	M7363	LV	Black6	WT		4
10	d8 Het	LV	RBM20	Het		4
11	M7271	LV	RBM20	Hom		4
12	BL6 TC	TC	BL6	WT		4

Uncropped titin isoform analysis of Figure 1B, at 8 weeks

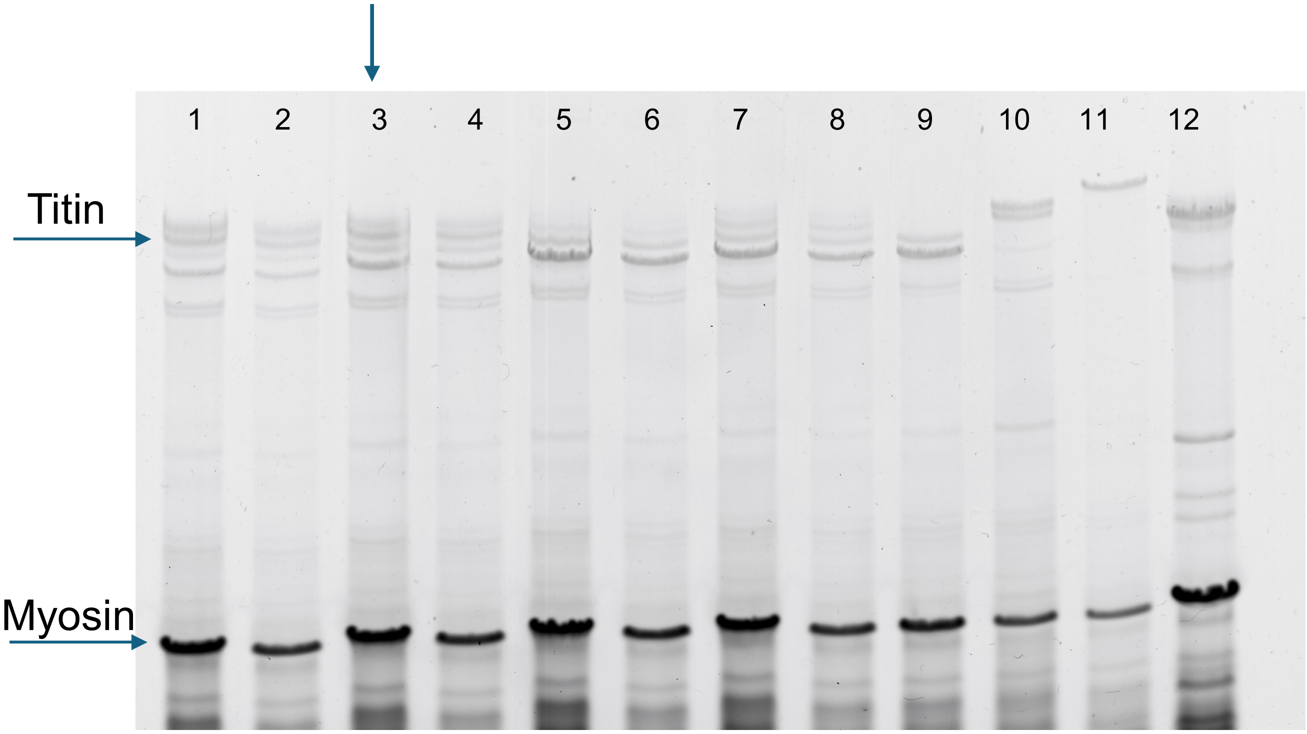

Lane	Sample ID	Muscle	Strain	Genotype	Treatment	Vol (uL)
1	100-2210 DLP	LV	Black6N	WT	ASO 25mg/kg 8 doses	9
2	100-2210 DLP	LV	Black6N	WT	ASO 25mg/kg 8 doses	4.5
3	100-2210 DRP	LV	Black6N	WT	ASO 25mg/kg 8 doses	9
4	100-2210 DRP	LV	Black6N	WT	ASO 25mg/kg 8 doses	4.5
5	100-2213 RP	LV	Black6N	WT	ASO 10mg/kg 8 doses	9
6	100-2213 RP	LV	Black6N	WT	ASO 10mg/kg 8 doses	4.5
7	100-2213 DLP	LV	Black6N	WT	ASO 10mg/kg 8 doses	9
8	100-2213 DLP	LV	Black6N	WT	ASO 10mg/kg 8 doses	4.5
9	M7363	LV	Black6	WT		4
10	d8 Het	LV	RBM20	Het		4
11	M7271	LV	RBM20	Hom		4
12	BL6 TC	TC	BL6	WT		4

Uncropped RBM20 western blot of Figure 2B

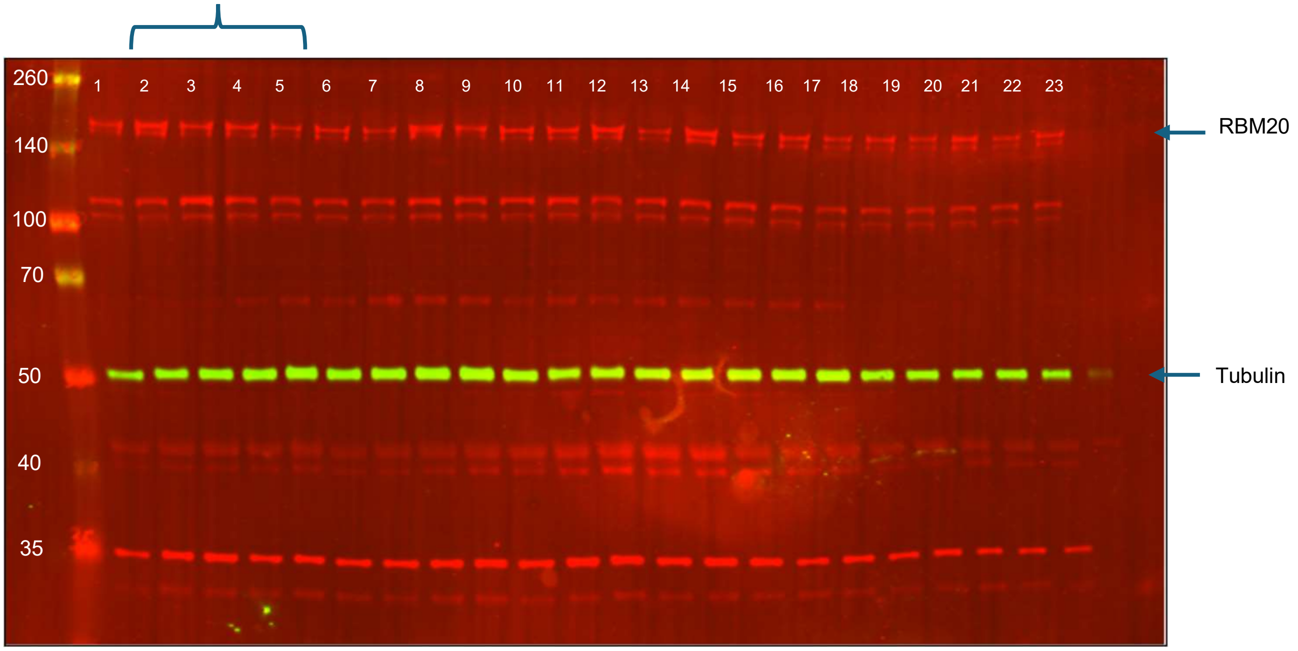

Lane	Sample ID	Muscle	Strain	Diet	Treatment	Total/Tubulin
1	86-4878LP	LV	C57BL/6N	NIH diet		0.325815603
2	100-3598 RP	LV	C57BL/6N	Low fat diet	PBS	0.466512702
3	100-3598 LP	LV	C57BL/6N	Low fat diet	ASO 25mg/kg	0.214159292
4	100-3597 LP	LV	C57BL/6N	Two Hit	PBS	0.247244094
5	100-3596 LP	LV	C57BL/6N	Two Hit	ASO 25mg /kg	0.142647059
6	100-3598 DRP	LV	C57BL/6N	Low fat diet	PBS	0.225462185
7	100-3598 NP	LV	C57BL/6N	Low fat diet	ASO 25mg /kg	0.141397059
8	100-3597 RP	LV	C57BL/6N	Two Hit	PBS	0.234375
9	100-3596 DLP	LV	C57BL/6N	Two Hit	ASO 25mg /kg	0.13172619
10	100-8579 LP	LV	C57BL/6N	Low fat diet	PBS	0.219172932
11	100-8579 RP	LV	C57BL/6N	Low fat diet	ASO 25mg /kg	0.286314655
12	100-3597 DLP	LV	C57BL/6N	Two Hit	PBS	0.370221328
13	100-3597 DRP	LV	C57BL/6N	Two Hit	ASO 25mg /kg	0.1375
14	100-8580 DLP	LV	C57BL/6N	Low fat diet	PBS	0.358333333
15	100-8580 LP	LV	C57BL/6N	Low fat diet	ASO 25mg /kg	0.187730496
16	100-8580 NP	LV	C57BL/6N	Two Hit	PBS	0.186917293
17	100-8578 LP	LV	C57BL/6N	Two Hit	ASO 25mg /kg	0.093055556
18	100-8579 NP	LV	C57BL/6N	Low fat diet	PBS	0.158208955
19	100-8580 RP	LV	C57BL/6N	Low fat diet	ASO 25mg /kg	0.122285714
20	100-8578 RP	LV	C57BL/6N	Two Hit	PBS	0.253986928
21	100-8578 DLP	LV	C57BL/6N	Two Hit	ASO 25mg /kg	0.128199052
22	86-4878LP	LV	C57BL/6N	NIH diet		0.276229508
23	M7270	LV	RBM20		Hom	

Uncropped titin isoform analysis of Figure 2C

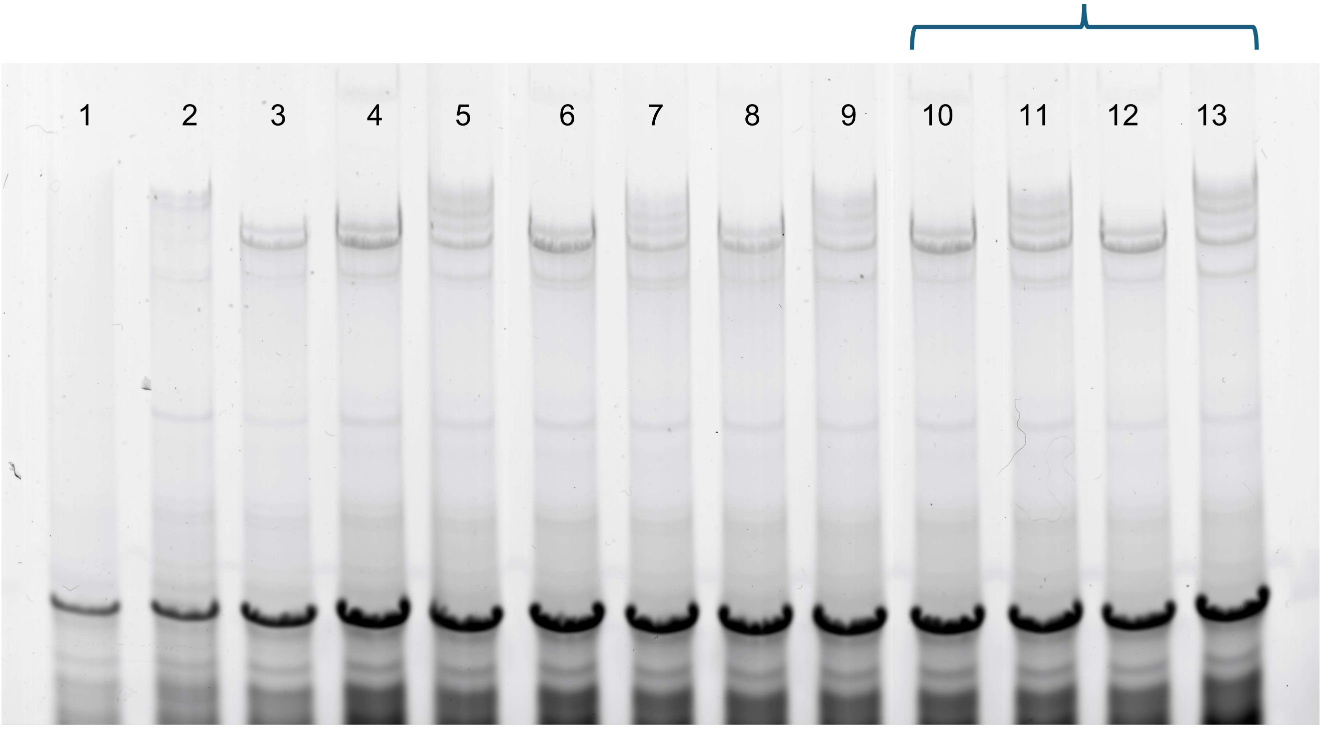

Lane	Sample ID	Muscle	Strain	Genotype	Treatment	Vol (uL)
1	M7271	LV	RBM20	Hom		5
2	d8 Het	LV	RBM20	Het		5
3	M7363	LV	Blac6	WT		5
4	98-3730DLP	LV	Black6N	WT	2Hit, PBS	9
5	98-2447DLP	LV	Black6N	WT	2Hit, ASO 25mg/kg 6 doses	9
6	98-3731RP	LV	Black6N	WT	Control Diet, PBS	9
7	98-2448RP	LV	Black6N	WT	Ctrl, ASO 25mg/kg 6 doses	9
8	98-3730RP	LV	Black6N	WT	2Hit, PBS	9
9	98-2447RP	LV	Black6N	WT	2Hit, ASO 25mg/kg 6 doses	9
10	98-3731LP	LV	Black6N	WT	Control Diet, PBS	9
11	98-2448LP	LV	Black6N	WT	Ctrl, ASO 25mg/kg 6 doses	9
12	98-3730LP	LV	Black6N	WT	2Hit, PBS	9
13	98-2447LP	LV	Black6N	WT	2Hit, ASO 25mg/kg 6 doses	9
